# Local Food Interventions for the Management of Moderate Acute Malnutrition (MAM) among Children Aged 6 to 59 Months: A Systematic Review and Non-inferiority Meta-Analysis

**DOI:** 10.1016/j.advnut.2026.100707

**Published:** 2026-08-07

**Authors:** Eskeziaw Abebe Kassahun, Hamid Yimam Hassen, Alinoor Mohamed Farah, Yakob Desalegn Nigatu, Debritu Nane, Emily C Keats, Genet Kiflemariam Paulos, Tesfamichael Awoke Sissay, Kemeria Barsenga, Aweke Kebede, Robert Ackatia-Armah, Paluku Bahwere, Samson Gebremedhin, Seifu Hagos Gebreyesus

**Affiliations:** 1 Department of Nutrition and Dietetics, School of Public Health and College of Health Science, Addis Ababa University, Addis Ababa, Ethiopia; 2 Department of Family Medicine & Population Health, Faculty of Medicine and Health Sciences, University of Antwerp, Antwerp, Belgium; 3 Environmental Intelligence Unit, Flemish Institute for Technological Research (VITO), Mol, Belgium; 4 School of Public Health, College of Health Science and Medicine, Wolaita Sodo University, Sodo, Ethiopia; 5 Institute for Global Nutrition, University of California Davis, Davis, CA, United States; 6 Department of International Health, Johns Hopkins Bloomberg School of Public Health, Baltimore, MD, United States; 7 World Food Program, Addis Ababa, Ethiopia; 8 Independent Researcher, Brussels, Belgium

**Keywords:** moderate acute malnutrition, locally available foods, locally processed foods, specialized nutritious foods, non-inferiority meta-analysis, child nutrition, community-based management of acute malnutrition

## Abstract

Moderate acute malnutrition (MAM) remains a major public health challenge among children aged 6 to 59 mo. Specialized nutritious foods (SNFs) are the standard intervention for the management of MAM; however, their use is constrained by supply chain disruptions, high costs, limited local production, and sustainability challenges, highlighting the need for context-specific alternatives. We conducted a systematic review and non-inferiority meta-analysis to compare local food interventions with SNFs for recovery rate and weight gain among children with MAM. We searched Medical Literature Analysis and Retrieval System On Line, Excerpta Medica dataBASE, Cochrane Central Register of Controlled Trials, Web of Science, ProQuest, and grey literature sources. Randomized controlled trials and quasi-experimental studies comparing local food interventions with SNFs were included. Local food interventions were categorized into the following 2 groups: locally available foods (LAFs), prepared from local ingredients using household or community methods, and locally processed foods (LPFs), produced from locally sourced ingredients through processing and fortification. To demonstrate non-inferiority, the predefined non-inferiority margins were set at 10% for recovery rate and 1.2 g/kg/d for weight gain. Non-inferiority was established when the lower bound of the 95% confidence interval (CI) remained above the corresponding margin. A network meta-analysis was performed to compare the relative effectiveness of LAFs, LPFs, fortified blended food, and ready-to-use supplementary food. Twenty-one studies met the eligibility criteria, and 13 were included in the meta-analyses. For recovery rate, LAFs were inferior to SNFs [risk difference (RD): −0.21; 95% CI: −0.33, −0.08], whereas LPFs were non-inferior (RD: −0.03; 95% CI: −0.09, 0.03). For weight gain, both LAFs [mean difference (MD): −0.33; 95% CI: −0.56, −0.11] and LPFs (MD: −0.32; 95% CI: −0.43, −0.21) resulted in lower weight gain than SNFs but met the predefined non-inferiority criterion. In conclusion, LPFs can serve as effective alternatives to SNFs for managing MAM. Further research is needed to optimize the formulation and effectiveness of LAFs for MAM management.

This review was registered at PROSPERO as CRD42024566607.

**Statement of Significance**

To our knowledge, this review provides the first comprehensive comparison of the effectiveness of locally available foods and locally processed foods for the management of moderate acute malnutrition in children aged 6 to 59 mo. Locally processed foods met the predefined non-inferiority criteria for both recovery rate and weight gain compared with specialized nutritious foods, whereas locally available foods did not meet the criterion for recovery rate, highlighting the need to optimize their formulation and nutritional quality to improve treatment outcomes.

## Introduction

Moderate acute malnutrition (MAM) is a major public health concern worldwide among children <5 years of age. It is defined as a weight-for-height *z*-score (WHZ) between −3 and <−2 SD and/or a mid-upper arm circumference (MUAC) between 115 and <125 mm, without bilateral pitting edema [1]. MAM is associated with increased morbidity, impaired growth and development, and a higher risk of progression to severe acute malnutrition (SAM) [2]. Globally, an estimated 47 million children suffer from acute malnutrition, of whom ∼32.7 million have MAM [3]. Although SAM receives greater funding support and clinical attention because of its higher mortality risk, MAM remains highly prevalent, particularly in low- and middle-income countries (LMICs) [4]. Effective management of MAM is essential to prevent progression to SAM, reduce mortality risk, and support sustained nutritional recovery.

Global strategies for MAM management have primarily relied on specialized nutritious foods (SNFs), including ready-to-use supplementary foods (RUSFs) and fortified blended foods (FBFs) [5]. These products are widely used in community-based nutrition programs and have been effective at improving nutritional recovery. However, their use is constrained by global shortages, supply chain disruptions, high costs, limited local production, and sustainability concerns [[6], [7], [8]]. Recent reductions in international donor funding further highlight the vulnerability of reliance on imported nutritional products and underscore the need for sustainable, locally adaptable alternatives for MAM management in LMICs [9]. In response to these challenges, increasing attention has been given to locally sourced or locally prepared food-based interventions as potential alternatives to SNFs. Such approaches may improve sustainability, strengthen local food systems, enhance community resilience, and increase the cultural acceptability of nutritional interventions [10]. The 2023 WHO guidance emphasizes the use of locally available, nutrient-dense foods for children with MAM and recommends reserving SNFs for high-risk children with comorbidities, poor recovery, or maternal health concerns that could negatively influence child outcomes [11]. Nevertheless, evidence on the comparative effectiveness of local food-based interventions remains limited and fragmented.

Previous evidence syntheses have evaluated nutritional interventions for MAM management; however, important gaps remain. A 2013 Cochrane systematic review examined dietary interventions for MAM in children in LMICs. It encompassed specially formulated supplementary foods, lipid-based nutrient supplements, blended foods, complementary food supplements, and locally prepared diets. Nevertheless, the review did not separately classify or specifically analyze local food-based interventions as a distinct category [12]. A subsequent 2020 Cochrane review examined food voucher interventions among children aged 6 to 59 mo, older children, and females [5]. However, the 4 related studies in this review did not clearly specify the types of foods purchasable through voucher programs for MAM management, limiting its applicability to the scope of our review and raising concerns about the nutritional adequacy of purchased foods for effective MAM management.

Thus, to address the limitations of the current SNF-dependent approaches, it is important to evaluate the effectiveness and non-inferiority of locally sourced foods and establish the minimum dietary standards required as an alternative strategy for MAM management in children. Establishing evidence on locally adaptable dietary alternatives may support sustainable nutritional strategies and contribute to achieving the World Health Assembly target of reducing childhood wasting to <3% by 2030 [13]. Therefore, this study aimed to comprehensively synthesize evidence comparing locally sourced or produced food interventions with SNFs for the management of MAM, hypothesizing non-inferior outcomes.

## Methods

### Design and protocol registration

This review was reported in accordance with the 2020 PRISMA guidelines [14]. A completed PRISMA checklist is available in Supplemental Table 1. The protocol for this review was registered with PROSPERO as CRD42024566607. During the review process, we refined the intervention classification and analytical framework to better capture the heterogeneity of the included studies. Specifically, interventions were grouped into locally available food (LAF) and locally processed food (LPF) categories, and a non-inferiority framework was applied. These adjustments, made during data extraction, did not alter the primary research question or overall study objectives.

### Search strategy

We searched for relevant published articles in international electronic databases, including MEDical Literature Analysis and Retrieval System On Line, Excerpta Medica dataBASE, Cochrane Central Register of Controlled Trials, ProQuest, and Web of Science from inception to 9 November, 2024. We updated the search on 4 July, 2025. We conducted an extensive grey literature search using sources such as Google Scholar, clinicaltrials.gov, the World Food Programme (WFP) organizational webpage, and the WHO International Clinical Trials Registry Platform. The search used a comprehensive range of relevant terms applied to titles, abstracts, and keywords to ensure the inclusion of all potentially eligible articles related to LAF interventions for MAM. The search was unrestricted by date, language, study design, or publication status to maximize the inclusion of relevant studies. We also performed citation tracking of included articles and reviewed reference lists to identify any eligible studies missed in the initial database and grey literature searches. Detailed database search strategies are available in Supplemental Table 2.

### Study selection process

Articles identified through database searches and manual searches were exported to the reference management software EndNote (Clarivate Analytics). Duplicates were removed, and titles and abstracts were screened against the predefined eligibility criteria by EAK and GKP. Disagreements regarding study eligibility were resolved through discussion between EAK and GKP, and when consensus could not be reached, HYH acted as the third reviewer and made the final decision. When essential study information was missing, the corresponding authors were contacted by e-mail to request clarification or additional data.

### Eligibility criteria

#### Study population

Children aged 6 to 59 mo with MAM, defined by ≥1 of the following criteria:

1. WHO Growth Standards: WHZ between <−2 and ≥−3 SD, and/or MUAC ≥115 mm and <125 mm, in the absence of bilateral pitting edema.
2. National Center for Health Statistics (NCHS) reference: weight-for-height (WFH) between 70% and 80% of the median, MUAC between 110 mm and <125 mm, and no bilateral pitting edema.

#### Intervention

Locally sourced foods for the management of MAM were classified into the following 2 main subtypes:

1. LAFs were defined as foods sourced directly from the local environment or prepared from locally available ingredients, such as cereals, legumes, nuts, and seeds, using traditional household or community preparation practices without advanced processing or nutritional enhancement. These foods were consumed in their natural or customary form, without micronutrient fortification or addition of specialized ingredients intended to increase therapeutic nutritional value. Interventions that did not involve food consumption but that promoted or encouraged the use, preparation, or consumption of locally available household foods for the dietary management of MAM (e.g., through nutritional counseling) were also classified as LAFs.
2. LPFs were defined as foods made from locally sourced ingredients (e.g., cereals, legumes, nuts, and seeds) that underwent small-scale processing and formulation in laboratory or industrial facilities. These foods had added micronutrient premixes, minerals, or other nutrient-enhancing ingredients obtained from specialized manufacturers or food-processing organizations, such as Nutriset, the WFP, or others, to improve nutrient density and therapeutic nutritional value for the management of MAM. LPFs, therefore, referred to locally derived foods that were fortified or nutritionally enhanced beyond their original composition.

#### Comparison

Control groups receiving SNFs defined as preformulated, fortified nutritional products specifically designed to prevent and treat MAM in children. SNFs are typically procured or distributed by organizations such as the WFP, Nutriset, or other certified food-processing organizations. These products were classified as follows:

1. FBF: blended food supplements such as Corn-Soy Blend (CSB, CSB+, and CSB++) and similar formulations (e.g., wheat-soy flour). They are often mixed with ingredients such as sugar, oil, and legumes and are typically prepared as porridge or soup before being fed to children.
2. RUSF: lipid-based, energy-dense food products designed for direct consumption without the need for cooking or preparation (e.g., Plumpy’Sup and high-energy biscuits). RUSFs are characterized by their high lipid content and adherence to globally recognized nutritional and safety standards.

#### Outcomes

Studies were included if they reported ≥1 of the following predefined primary outcomes: recovery rate, weight gain, height gain, WHZ change, height-for-age *z*-score (HAZ) change, MUAC gain, time to recovery, death rate, or deterioration to SAM.

#### Types of studies

Original research studies employing randomized controlled trials (RCTs) or controlled quasi-experimental designs were included.

### Full-text screening

**FIGURE 1:**
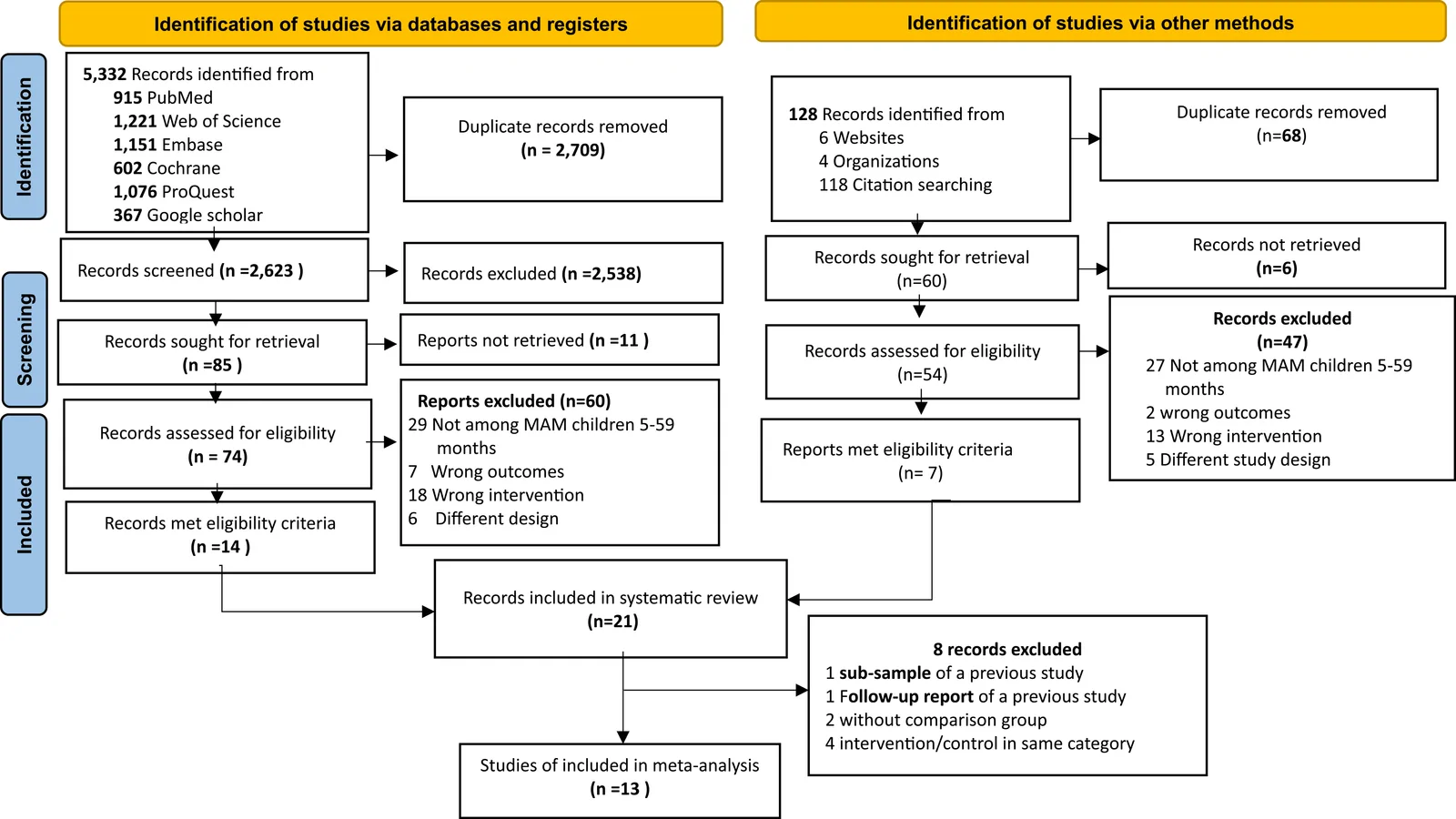
PRISMA 2020 flow chart illustrating the article search and selection process. MAM, moderate acute malnutrition.

After title and abstract screening, the included references were exported to Rayyan (https://www.rayyan.ai/), a web-based artificial intelligence tool [15], to support full-text review for final eligibility assessment. EAK and GKP independently screened the full texts for eligibility. Disagreements were resolved through discussion between EAK and GKP, and unresolved disagreements were adjudicated by HYH. Studies with multiple publications were treated as a single study. We contacted authors by e-mail twice to obtain missing information. Conference abstracts and articles with unavailable full texts were excluded after 2 unsuccessful attempts to contact the authors. All reasons for exclusion were documented, and the study selection process is detailed in the PRISMA flow chart (Figure 1).

### Data extraction and management

A standardized data extraction form was developed in Microsoft Excel in alignment with the study objectives and eligibility domains. The form was pretested for consistency and refined before formal data extraction to ensure accuracy and reliability. EAK independently extracted data from all eligible studies, including study design, population characteristics, sample size, intervention details, comparator information, follow-up duration, frequency of follow-up assessments, and outcome measures. GKP independently verified all extracted data for accuracy and completeness. For studies with multiple intervention arms evaluating different local food-based treatments, data were extracted separately for each eligible comparison to facilitate detailed comparative analyses. Any discrepancies between EAK and GKP were resolved through discussion and consensus. When consensus could not be reached, HYH adjudicated the disagreement and made the final decision. The finalized dataset was then exported to R statistical software, version 4.3.2 (R Foundation for Statistical Computing, Vienna, Austria) for further processing and meta-analysis.

### Risk-of-bias assessment

The quality of the included studies was independently assessed by EAK and GKP at the outcome level using standardized risk-of-bias assessment tools. Disagreements were resolved through discussion between EAK and GKP, and unresolved discrepancies were adjudicated by HYH. For RCTs, the revised Cochrane Risk of Bias tool (RoB 2) [16] was employed, categorizing studies as having “low,” “some concerns,” or “high” risk of bias based on the domains of the randomization process, deviations from intended interventions, missing outcome data, measurement of outcomes, and selection of the reported result. For nonrandomized studies, the Risk of Bias in Non-randomized Studies of Interventions (ROBINS-I) tool [17] was used. This tool assesses bias across multiple domains, including confounding, selection of participants, classification of interventions, deviations from intended interventions, missing data, measurement of outcomes, and selection of reported results, categorizing studies as having “low,” “moderate,” “serious,” or “critical” risk of bias.

### Data synthesis

Descriptive statistics were used to summarize study characteristics, including study design, intervention type, and comparison group. Data are presented in tabular form for comparison purposes, highlighting study details including study country, year of study, intervention duration, and outcomes reported, among others. Studies were assessed for meta-analysis eligibility based on outcome measurement, type of local food intervention and control, and population. Meta-analyses were conducted for outcomes reported in ≥2 studies, and the remaining findings were synthesized narratively.

We performed a random-effects meta-analysis for each outcome separately. For continuous outcomes, such as weight gain, MUAC, WHZ, HAZ, and time to recovery, a mean difference (MD) with 95% confidence intervals (CIs) was used to summarize the pooled effect. When the SDs or SEs for differences were not available, we imputed them using other reported parameters based on the Cochrane guidelines [18]. For dichotomous outcomes, such as recovery rate, deterioration rate, and death rate, a risk difference (RD) with 95% CIs was used. Non-inferiority of the local food intervention compared with the control group was assessed by comparing the lower and upper bounds of the 95% CI for the MDs and RDs with predefined margins [19]. Margins of 10% for recovery rate, 1.2 g/kg/d for weight gain, and 14 d for time to recovery were established based on previous research [[20], [21], [22]] and expert consultation. For recovery rate and weight gain, non-inferiority was concluded when the lower bound of the 95% CI remained within the predefined margin, whereas for time to recovery, the upper bound of the 95% CI remained below the predefined margin.

A network meta-analysis (NMA) was employed to enable simultaneous comparisons among all relevant interventions. This approach integrated both direct evidence from head-to-head trials and indirect evidence derived from trials sharing common interventions, allowing for the estimation of relative treatment effects and the development of an intervention hierarchy. The resulting synthesis directly informs evidence-based decision making for the management of MAM [23].

The magnitude of heterogeneity between included studies was quantitatively assessed using the index of heterogeneity (*I*^2^ statistic) with 95% CIs. *I*^2^ values of 25%, 50%, and 75% are assumed to represent low, medium, and high heterogeneity, respectively. The significance of heterogeneity was determined by the *P* value of the *I*^2^ statistics, with a *P* value < 0.05 considered evidence of significant heterogeneity [24]. In cases where significant heterogeneity is detected, sensitivity analyses were conducted to assess the robustness of the results. Subgroup analyses were performed based on the types of local food interventions and control groups to explore any possible differences.

Funnel plots were used to visually evaluate the symmetry of study effects against SEs for each outcome. Publication bias was then explored visually using the funnel plots and through Egger’s test of asymmetry, conducted under a random-effects model.

## Results

The database search resulted in the retrieval of 5332 records (Figure 1). After duplicate removal, 2623 records underwent title and abstract screening, resulting in 85 articles being assessed for eligibility. Of these, 14 met the eligibility criteria. An additional 128 records were identified through grey literature sources, with 7 retained for inclusion. We attempted to contact the authors of 6 conference abstracts, but no responses were received, and these studies were consequently excluded. Overall, 21 articles were included in the review [21,22,[25], [26], [27], [28], [29], [30], [31], [32], [33], [34], [35], [36], [37], [38], [39], [40], [41], [42], [43]].

Of the 21 studies reviewed, 8 were excluded from the meta-analysis based on predefined eligibility criteria. These studies were excluded because they represented subsample analyses of previously included studies [35], were follow-up reports of primary studies already included in the review [27], lacked an eligible comparison group [32,40], or compared intervention and control groups that were classified within the same intervention category, such as FBF vs. FBF or RUSF vs. RUSF [31,39,41,42]. Consequently, 13 studies [21,22,25,26,29,30,33,34,[36], [37], [38], [39],^43^] were included in the various meta-analyses. Furthermore, 3 studies comparing FBF and RUSF groups [21,34,37] were specifically considered for NMA (Figure 1).

### Study characteristics

Of the 21 included studies, the majority (66.7%) were conducted in Sub-Saharan Africa (SSA): 4 studies were conducted in Malawi [21,34,42,43], 2 each were conducted in Uganda [26,30] and Mali [25,35], and 1 each was conducted in Ethiopia [22], Burkina Faso [38], Benin [32], Ghana [31], and Tanzania [33]. The South-East Asia Region contributed 5 studies (23.8%), with 1 from Indonesia [40] and 4 from Bangladesh [27,28,36,41]. The remaining single studies were conducted in Brazil (4.8%) [39] and Iran (4.8%) [29].

The studies included in this analysis were published between 1992 and 2024. Among the 19 RCTs, 7 were cluster randomized [25,26,30,35,37,38,41], and 12 were individually randomized [21,22,[27], [28], [29],^31^,^33^,^34^,^36^,^39^,^42^,^43^]. In addition, 2 studies applied quasi-experimental or nonrandomized controlled designs [32,40].

Regarding study settings, 14 studies were conducted in rural areas [21,22,25,26,[30], [31], [32], [33], [34], [35],^38^,[41], [42], [43]], 5 in urban areas [[27], [28], [29],^36^,^39^], and 2 in both urban and rural areas [37,40]. In total, 13 studies used 2 study arms [22,[26], [27], [28], [29], [30], [31], [32],^36^,^37^,[39], [40], [42]], 6 studies used 3 arms [21,33,[34], [38], [41],^43^], and 2 used 4 arms [25,35]. Seven studies provided results on >2 comparisons [21,[33], [34], [35],^38^,^43^]. All studies enrolled children aged 6 to 59 mo, with variations in the specific age ranges of eligible children. The characteristics of the included studies are summarized in Supplemental Table 3.

Among the 19 RCTs assessed, 11 were rated as having a low risk of bias [21,22,[25], [26], [27],^30^,[33], [34], [35], [36],^43^], 6 were judged to have some concerns [29,31,37,38,41,42], and 2 were identified as having a high risk of bias based on the RoB 2 tool [28,39]. Among the non-RCTs, 2 were found to have a high risk of bias according to the Cochrane ROBINS-I tool [32,40]. Details of risk of bias for individual studies and domains are provided in the supplementary material (Supplemental Table 4A–C).

### Interventions

The studies included in this review evaluated a range of local food interventions designed to treat MAM. LAFs included food supplements prepared from local ingredients [22,39,41], nutritional counseling [28,29,33,38], interventions in which no food supplement was provided during the trial period [43], and cashew nut seed flour [39]. LPFs included Sorghum Peanut Blend foods [26], Misola (cereal-legume blends), locally milled flours enriched with oil, sugar, and micronutrient powder [25,35], malted sorghum-based porridge [30], Microbiota-Directed Complementary Food No. 2 enriched with microbiota-active ingredients such as chickpea, soy flour, peanut paste, and green banana, along with oil, sugar, and micronutrients [27,36], fortified ready-to-eat biscuits [40], and FARIFORTI, a multi-ingredient infant flour composed of corn, malted sorghum, soybean, peanuts, baobab pulp, and dried fried fish [32]. Standard reference interventions included FBF and RUSF [21,31,34,37,42].

### Outcomes of the interventions

This systematic review investigated a range of outcomes to assess the effectiveness of local food interventions for the management of MAM. Recovery rate was the most frequently reported outcome and was reported in 11 studies. Of these, 7 studies consistently defined recovery as achieving a WHZ ≥−2 without bilateral pitting edema [21,26,29,30,33,34,38]. Three studies used a broader definition, considering recovery as achieving either a MUAC ≥12.5 cm and/or WHZ ≥−2 [22,25,31], and 2 studies defined recovery as WFH >85% of the NCHS median for 2 consecutive weeks [28,37]. Time to recovery was assessed in 5 studies [21,22,37,38,42], and risk of deterioration was assessed in 6 studies [21,33,34,37,38,42]. Anthropometric measurements were also widely evaluated, including HAZ in 7 studies [26,27,34,36,40,41,43], WHZ in 9 studies [21,26,27,29,34,36,38,40,42], weight gain in 17 studies [21,22,25,[27], [28], [29], [30], [31], [32], [33],[35], [36], [37], [38], [39],^42^,^43^], MUAC gain in 14 studies [21,22,25,27,31,33,[35], [36], [37], [38],[40], [41], [42], [43]], and height in 14 studies [21,22,25,27,29,30,33,35,36,[38], [39], [40],^42^,^43^].

### Effect of LAFs and LPFs on recovery rate

Table 1 summarizes meta-analyses comparing the effects of LAFs and LPFs against SNFs (RUSF or FBF) for the various outcomes.

**TABLE 1:** Pooled effects of locally available foods and locally processed foods on primary outcomes in the management of moderate acute malnutrition

Outcomes measure	Type of intervention	Control	Studies (*n*)	Pooled effect (95% CI)	*I*^2^ (95% CI)
Recovery rate	LAFs	Overall	6	−0.21 (−0.33, −0.08)^1^	91.0 (83.2, 96.5)
		LPFs	1	−0.40 (−0.62, −0.18)^1^	—
		FBF	3	−0.12 (−0.27, 0.03)	90.8 (76.1, 96.5)
		RUSF	3	−0.26 (−0.45, −0.07)^1^	96.0 (88.6, 98.6)
	LPFs	Overall	6	−0.03 (−0.09, 0.03)	79.3 (54.8, 90.5)
		FBF	3	0.01 (−0.04, 0.06)	56.8 (0.0, 85.7)
		RUSF	3	−0.12 (−0.18, −0.07)^1^	0.0 (0.0, 0.0)
Weight gain	LAFs	Overall	9	−0.33 (−0.56, −0.11)^1^	90.9 (84.9, 94.5)
		LPFs	2	−0.06 (−0.34, 0.21)	91.5 (70.1, 97.6)
		FBF	3	−0.30 (−0.52, −0.08)^1^	80.0 (47.0, 92.4)
		RUSF	2	−0.60 (−0.98, −0.22)^1^	91.0 (76.5, 96.5)
	LPFs	Overall	6	−0.32 (−0.43, −0.21)^1^	51.4 (0.0, 89.6)
		FBF	3	−0.26 (−0.38, −0.15)^1^	0.0 (0.0, 81.1)
		RUSF	3	−0.38 (−0.57, −0.19)^1^	69.9 (0.0, 91.2)
Time to recovery	LAFs	Overall	3	4.35 (−2.08, 10.8)	83.5 (50.0, 94.5)
		FBF	2	4.43 (−5.2, 13.96)	91.7 (71.2, 97.6)
		RUSF	1	4.20 (−1.54, 9.94)	—

Abbreviations: CI, confidence interval; FBF, fortified blended food; LAF, locally available food; LPF, locally processed food; MUAC, mid-upper arm circumference; RUSF, ready-to-use supplementary food; SNF, specialized nutritious food; WHZ, weight-for-height *z*-score.

1 Statistically significant difference.

Our analysis demonstrated that LAFs were associated with a lower recovery rate than SNFs, with an overall pooled RD of −0.21 (95% CI: −0.33, −0.08). In contrast, the pooled recovery rate for LPFs relative to SNFs was a pooled RD of −0.03 (95% CI: −0.08, 0.03).

**FIGURE 2:**
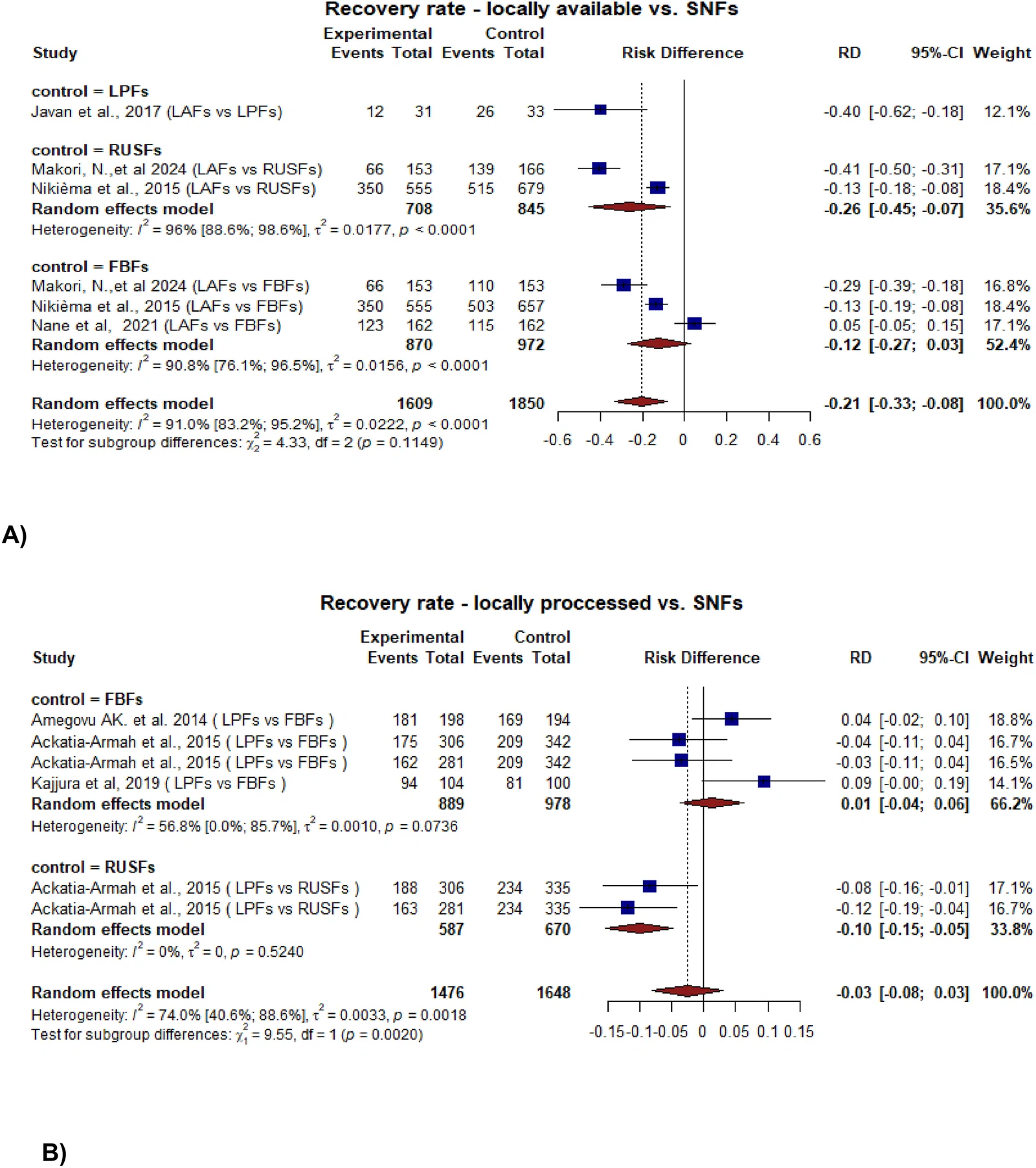
Forest plot of the effect of local food interventions vs. SNFs on recovery rates in children with moderate acute malnutrition. (A) Locally available foods (LAFs). (B) Locally processed foods (LPFs). CI, confidence interval; FBF, fortified blended food; RD, risk difference; RUSF, ready-to-use supplementary food; SNF, specialized nutritious food.

We conducted subgroup analyses comparing LAFs and LPFs with separate SNFs. Compared with RUSF, LAFs had a lower recovery rate (RD: −0.26; 95% CI: −0.45, −0.07), whereas the estimated effect relative to FBF was an RD of −0.12 (95% CI: −0.27, 0.03). For LPFs, the corresponding RDs were 0.01 (95% CI: −0.04, 0.06) relative to FBF and −0.10 (95% CI: −0.15, −0.05) relative to RUSF (Figure 2).

**FIGURE 3:**
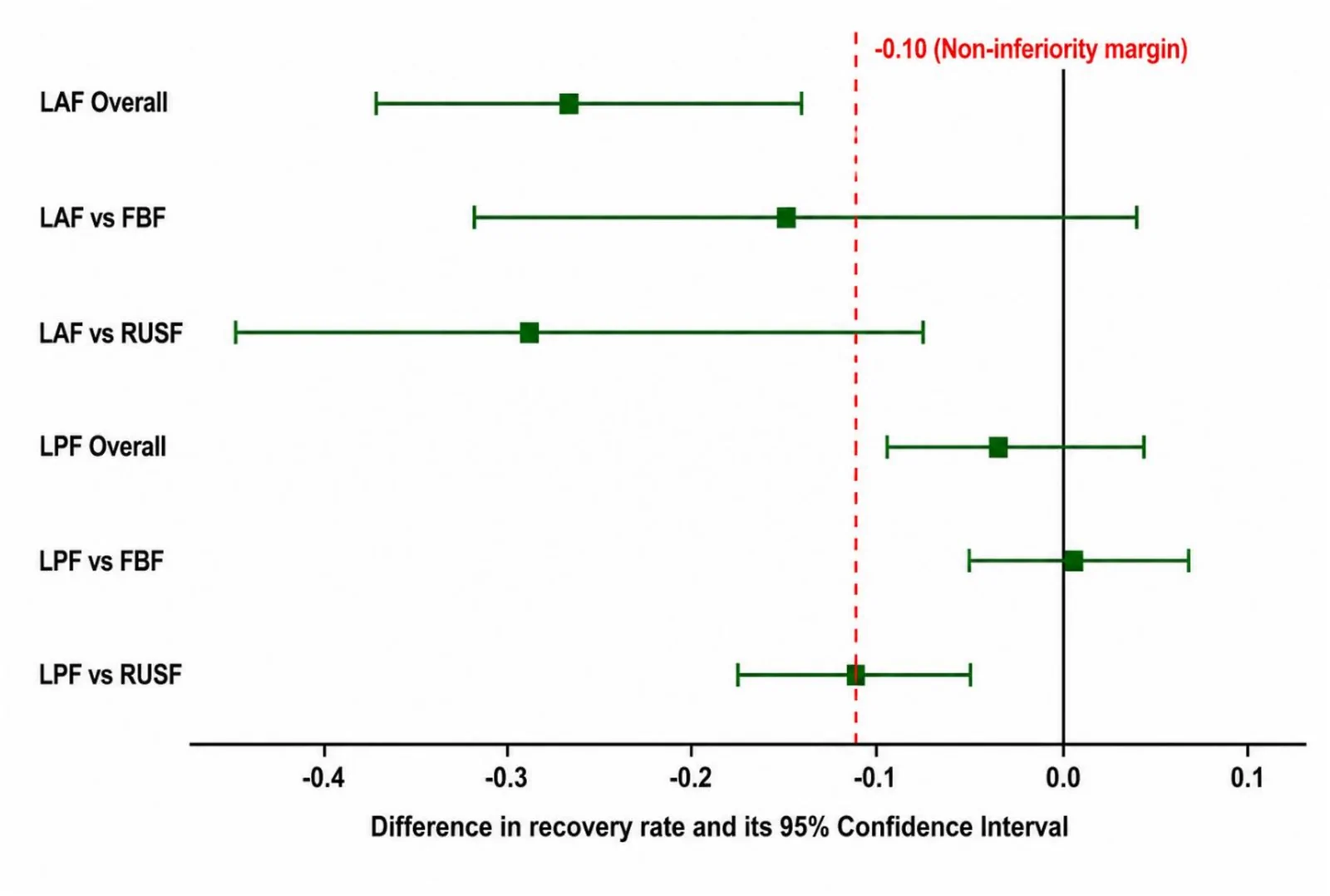
Difference in recovery rate with 95% confidence interval in LAFs and LPFs vs. SNFs. The plot displays risk difference in recovery rate for comparisons involving LAFs (overall LAFs, LAFs vs. FBF, LAFs vs. RUSF) and LPFs (overall LPFs, LPFs vs. FBF, LPFs vs. RUSF) with SNFs (RUSF and FBF). The overall risk difference in LAFs was generally inferior, and LPFs were non-inferior to the SNF groups. The solid vertical line at 0 indicates no difference in recovery rate. The dotted red line at −0.10 represents the predefined non-inferiority margin. FBF, fortified blended food; LAF, locally available food; LPF, locally processed food; RUSF, ready-to-use supplementary food; SNF, specialized nutritious food.

In summary, our analysis showed that LAFs were inferior to both RUSF and FBF, as the lower bound of the 95% CI for the RD fell below the predefined non-inferiority margin of −0.10. In contrast, LPFs generally demonstrated non-inferior recovery outcomes when compared with FBF. However, LPFs did not meet the strict non-inferiority criteria when compared against RUSF, although the effect estimate suggests broadly comparable recovery outcomes (Figure 3).

### Effect of LAFs and LPFs on weight gain

**FIGURE 4:**
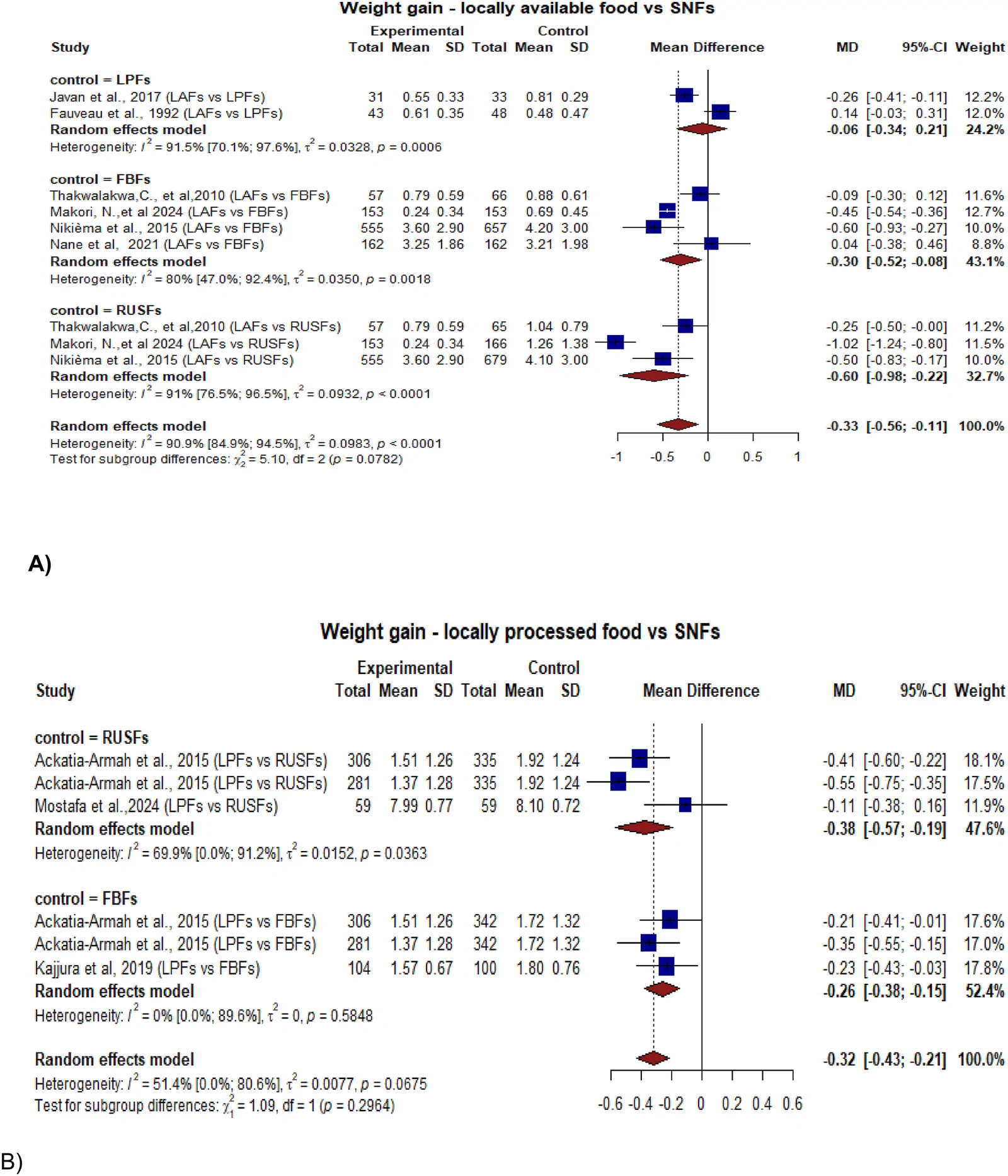
Forest plots indicating the effect of local food interventions vs. SNFs on weight gain in children with moderate acute malnutrition. (A) Locally available foods (LAFs). (B) Locally processed foods (LPFs). CI, confidence interval; FBF, fortified blended food; MD, mean difference; RUSF, ready-to-use supplementary food; SNF, specialized nutritious food.

**FIGURE 5:**
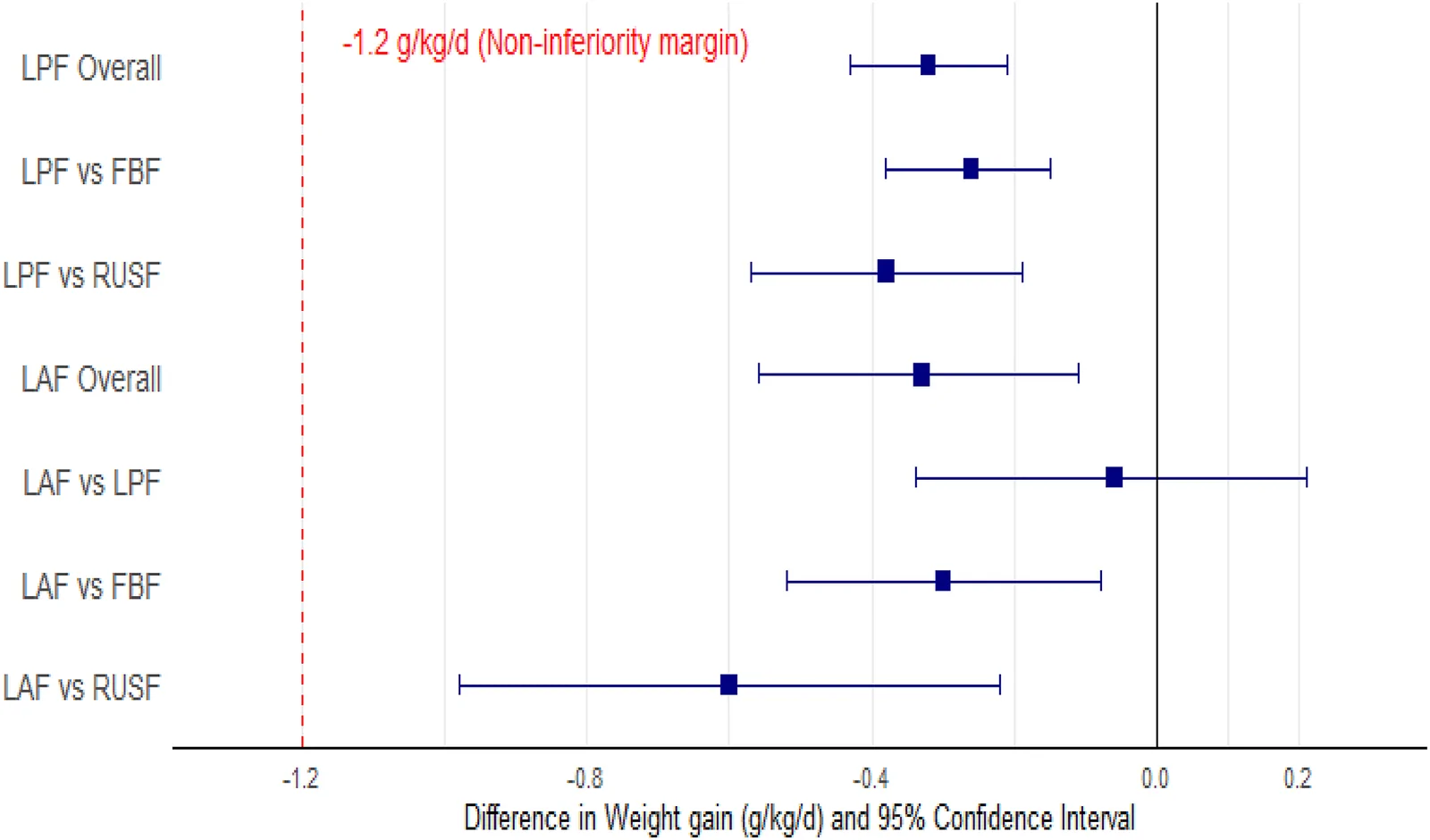
Difference in weight gain (grams per kilogram per day) with 95% confidence interval between LAFs and LPFs vs. SNFs. The plot displays the mean difference in weight gain for comparisons involving LAFs (overall LAFs, LAFs vs. FBF, LAFs vs. RUSF) and LPFs (overall LPFs, LPFs vs. FBF, LPFs vs. RUSF) with SNFs (RUSF and FBF). The overall mean difference in LAFs and LPFs was generally non-inferior to the SNF groups. The solid vertical line at 0 indicates no difference in weight gain. The dotted red line at −1.2 g/kg/d represents the predefined non-inferiority margin. FBF, fortified blended food; LAF, locally available food; LPF, locally processed food; RUSF, ready-to-use supplementary food; SNF, specialized nutritious food.

Our analysis showed that LAFs were associated with lower weight gain than SNF interventions, with a pooled MD of −0.33 g/kg/d (95% CI: −0.56, −0.11 g/kg/d). Similarly, LPFs were associated with lower weight gain than the SNF interventions, with a pooled MD of −0.32 g/kg/d (95% CI: −0.43, −0.21 g/kg/d) (Figure 4). Despite these differences, both LAFs and LPFs met the predefined non-inferiority criterion for weight gain, as the lower bounds of the 95% CIs remained above the non-inferiority margin of −1.2 g/kg/d (Figure 5).

### Effect of LAFs and LPFs on time to recovery

**FIGURE 6:**
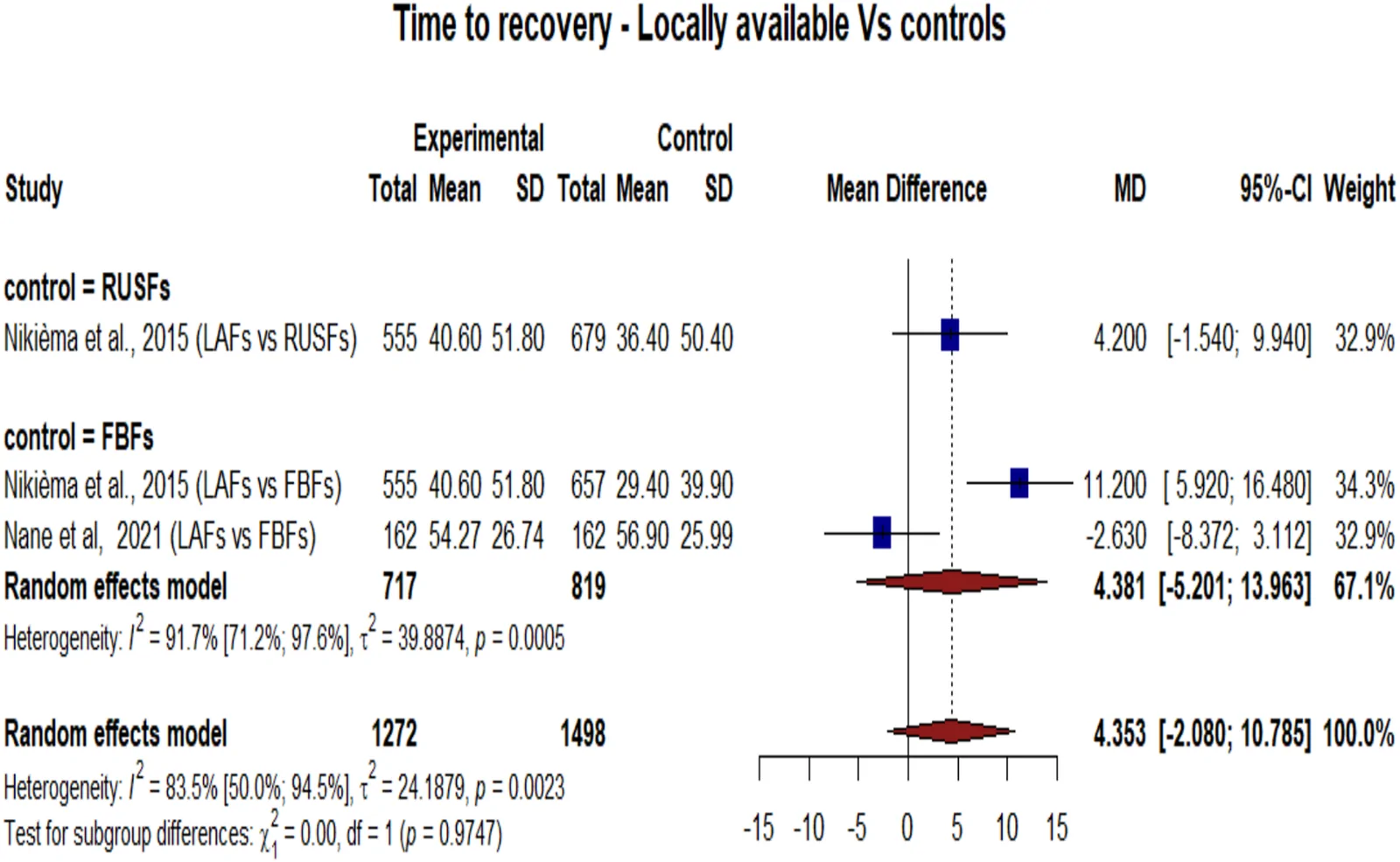
Forest plots indicating the effect of locally available food interventions vs. specialized nutritious foods on time to recovery in children with moderate acute malnutrition. FBF, fortified blended food; LAF, locally available food; MD, mean difference; RUSF, ready-to-use supplementary food.

**FIGURE 7:**
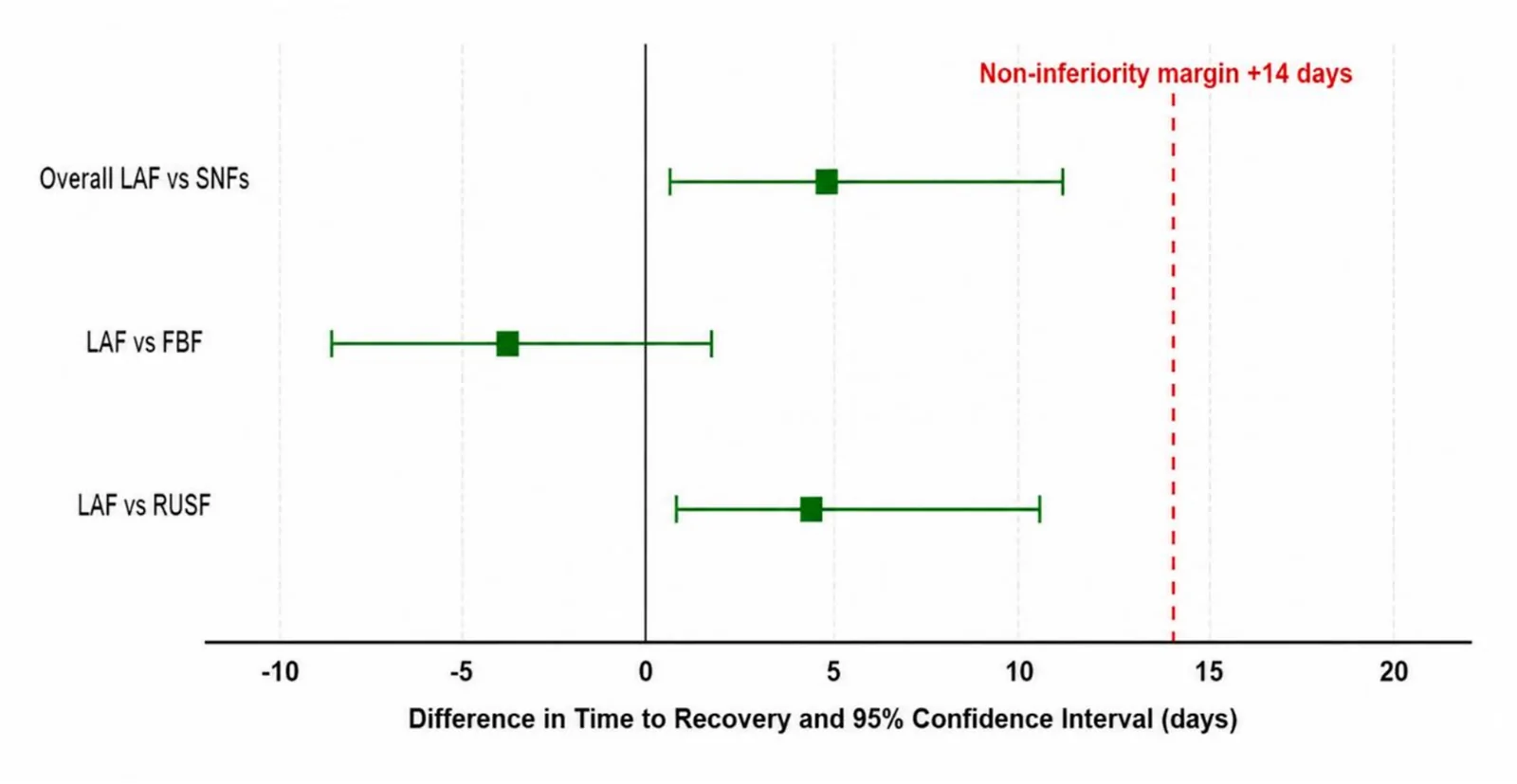
Difference in time to recovery in days with 95% confidence interval between LAFs and SNFs. The plot displays the mean difference in time to recovery for comparisons involving LAFs (overall LAFs, LAFs vs. FBF, LAFs vs. RUSF). The overall mean difference in LAFs was generally non-inferior to the SNF groups. The solid vertical line at 0 indicates no difference in days. The dotted red line at 14 d represents the predefined non-inferiority margin. FBF, fortified blended food; LAF, locally available food; MD, mean difference; RUSF, ready-to-use supplementary food; SNF, specialized nutritious food.

We examined the effect of LAFs on recovery time in children with MAM. Recovery time was reported only for LAF interventions; no studies reported this outcome for LPFs. Overall, LAF interventions were associated with a longer time to recovery than control groups, with an MD of 4.35 d (95% CI: −2.08, 10.79 d). Subgroup analysis showed that LAFs also had a longer recovery time than FBF controls (MD: 4.38; 95% CI: −5.20, 13.96) (Figure 6). Considering the predefined non-inferiority margin of 14 days, the upper bound of the 95% CI remained below this threshold, indicating that LAFs met the predefined non-inferiority criterion for time to recovery compared with FBF (Figure 7).

The effects of LAFs on other outcomes are presented in Supplemental Table 5. Forest plots for other outcomes are provided in the supplementary materials (Supplemental Figures 1–5).

### Network meta-analysis

We conducted an NMA to evaluate the comparative impact of different interventions on treatment outcomes of recovery rate and weight gain in children with MAM. The analysis revealed a network structure with varying degrees of direct evidence for different comparisons among LAFs, LPFs, FBF, and RUSF. Strong direct evidence was available for comparisons between FBF and RUSF and between LPFs and RUSF. In contrast, comparisons involving LAFs, such as LAFs vs. RUSF and LAFs vs. LPFs, relied primarily on indirect evidence because of limited direct comparisons in the included studies.

#### Recovery rate

**FIGURE 8:**
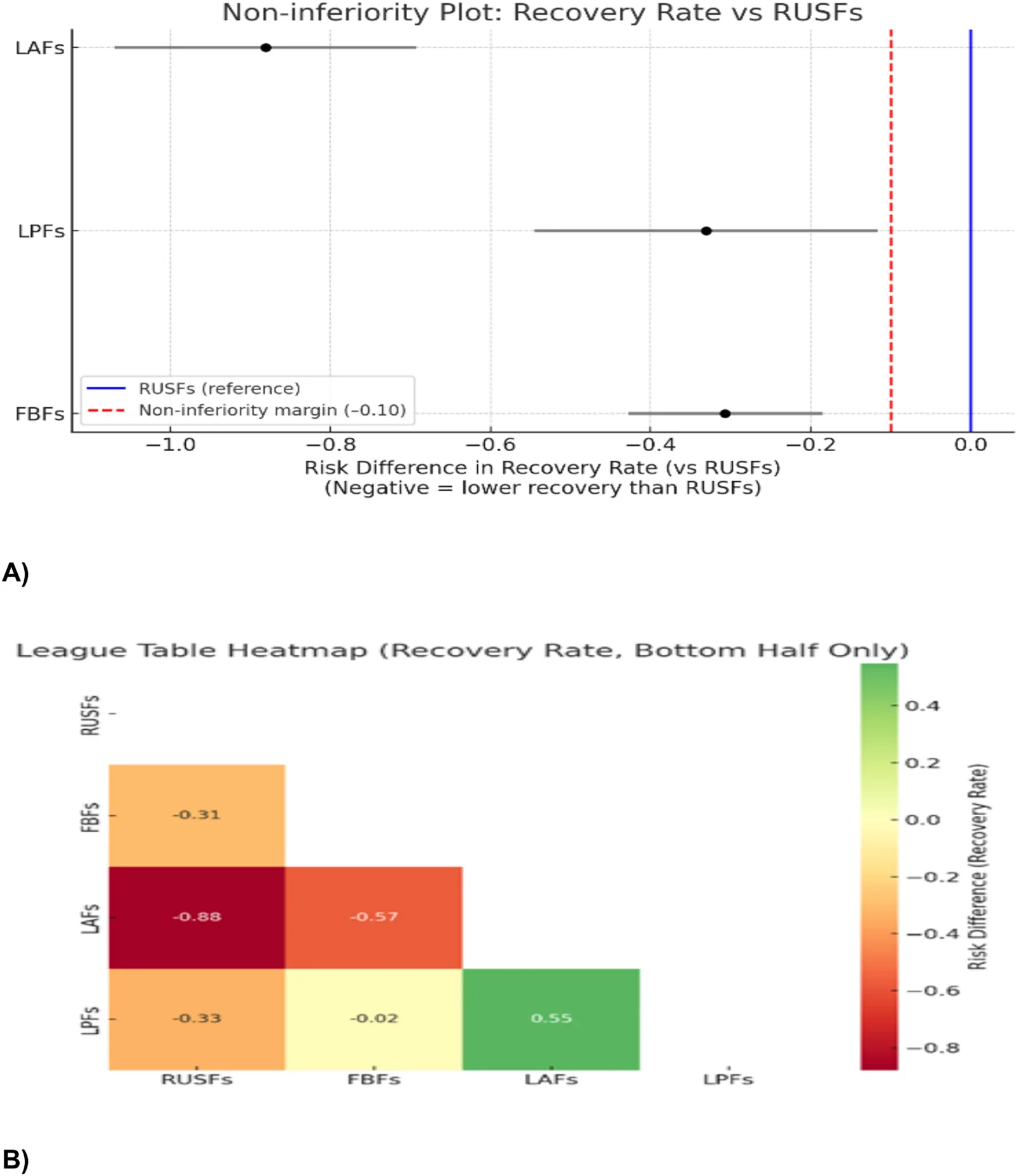
Network meta-analysis risk differences in recovery rate of interventions compared with specialized nutritious foods. (A) Forest plot showing risk difference in recovery rates for fortified blended foods (FBFs), locally processed foods (LPFs), and locally available foods (LAFs) compared with ready-to-use supplementary foods (RUSFs, reference). Points represent estimated risk differences, and horizontal lines indicate 95% confidence intervals. The blue vertical line marks the reference (RUSFs), and the red dashed line indicates the non-inferiority margin (−0.10). (B) League table summary of pairwise comparisons of recovery rates between different supplementary foods: RUSFs, FBFs, LAFs, and LPFs. Each cell shows risk difference in recovery rate, with negative values (red shades) indicating lower recovery rates for the row intervention compared with the column intervention, and positive values (green shades) indicating higher recovery rates.

For the recovery rate, 10 studies comprising 16 pairwise comparisons across 4 interventions were considered in the NMA. Compared with RUSF, all alternative interventions were associated with lower recovery rates: FBF (RD: −0.31; 95% CI: −0.43, −0.19), LPFs (RD: −0.33; 95% CI: −0.55, −0.12), and LAFs (RD: −0.88; 95% CI: −1.07, −0.69) (Table 2). In all cases, the CIs fell below the predefined non-inferiority threshold, indicating that none of these alternative interventions met the criterion for non-inferiority. This suggests that RUSF consistently outperformed the other interventions in terms of recovery rates (Figure 8).

**TABLE 2:** Pairwise comparison of risk difference in recovery rate for in the management of moderate acute malnutrition (recovery rate, risk difference, Δ = −0.10)

	RUSF	FBF	LAF
FBF	−0.31 (−0.43, −0.19)	—	—
LAF	−0.88 (−1.07, −0.69)	−0.57 (−0.76, −0.39)	—
LPF	−0.33 (−0.55, −0.12)	−0.02 (−0.23, 0.18)	0.55 (0.29, 0.81)

The efficacy endpoint is based on a 95% CI for risk difference. The prespecified non-inferiority margin is Δ = −0.10.

Abbreviations: FBF, fortified blended food; LAF, locally available food; LPF, locally processed food; RUSF, ready-to-use supplementary food; SNF, specialized nutritious food.

#### Weight gain

**FIGURE 9:**
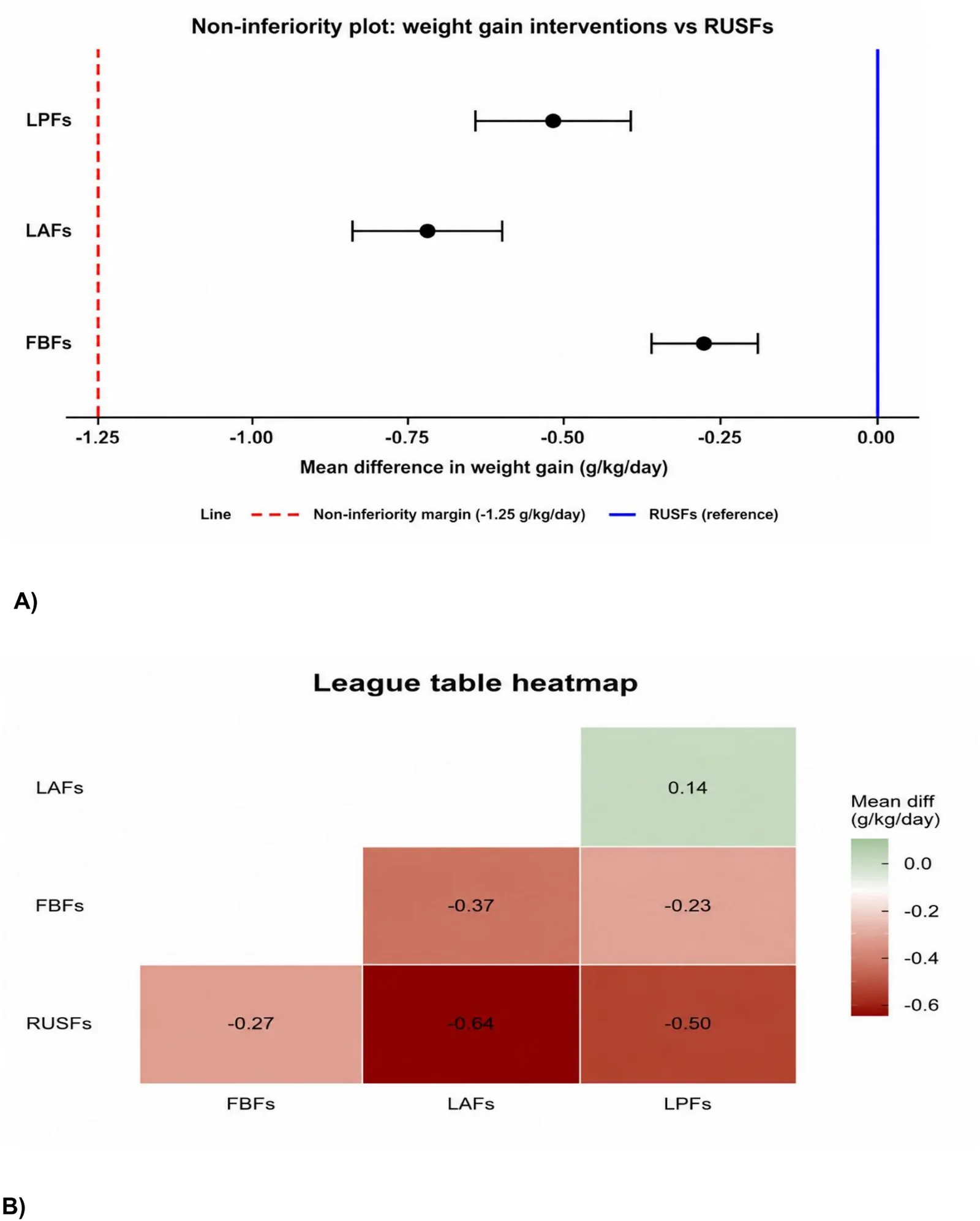
Network meta-analysis of risk difference in mean difference in weight gain for interventions compared with SNFs. (A) Forest plot of the mean difference in weight gain for fortified blended foods (FBFs), locally processed foods (LPFs), and locally available foods (LAFs) compared with ready-to-use supplementary foods (RUSFs, reference). Points represent estimated risk differences, and horizontal lines indicate 95% confidence intervals. The blue vertical line marks the reference (RUSFs), and the red dashed line indicates the non-inferiority margin (−1.2 g/kg/d). (B) League table summary of pairwise comparisons of mean weight gain between different supplementary foods: RUSFs, FBFs, LAFs, and LPFs. Each cell shows risk difference in recovery rate, with negative values (red shades) indicating lower weight gain for the row intervention compared to the column intervention, and positive values (green shades) indicating higher weight gain.

The NMA on weight gain included 11 studies encompassing 19 pairwise comparisons and 4 interventions: FBF, LAFs, LPFs, and RUSF as reference. Table 3 presents the results of these pairwise comparisons. Using a common-effects model and a non-inferiority margin of 1.2 g/kg/d, our analysis showed that FBF compared with RUSF had an MD of −0.27 (95% CI: −0.36, −0.18), whereas LPFs compared with RUSF had an MD of −0.50 (95% CI: −0.59, −0.41). In both cases, the CIs fell within the non-inferiority threshold, indicating that FBF and LPFs are non-inferior to RUSF. Similarly, for LAFs compared with RUSF, the MD was −0.64 (95% CI: −0.74, −0.55), with the CI also within the non-inferiority margin of −1.2 g/kg/d, demonstrating that LAFs are non-inferior to RUSF (Figure 9).

**TABLE 3:** Pairwise comparison of mean difference in recovery rate for the management of moderate acute malnutrition (weight gain, mean difference, Δ = −1.2 g/kg/d)

	RUSF	FBF	LAF
FBF	−0.27 (−0.36, −0.18)	—	—
LAF	−0.64 (−0.74, −0.55)	−0.37 (−0.44, −0.30)	—
LPF	−0.50 (−0.59, −0.41)	−0.23 (−0.31, −0.15)	0.14 (0.06, 0.22)

Efficacy endpoint is based on a 95% confidence interval for risk difference. The prespecified non-inferiority margin is Δ = −1.2 g/kg/d.

Abbreviations: FBF, fortified blended food; LAF, locally available food; LPF, locally processed food; RUSF, ready-to-use supplementary food; SNF, specialized nutritious food.

### Narrative synthesis

Higher weight gain was observed in children receiving LAF-based interventions in 6 studies [22,29,33,38,39,43], and 2 studies reported a shorter time to recovery than controls [22,26]. Greater MUAC gains were noted in 2 studies [22,33], with 1 reporting a higher recovery [22] than controls. One study found that children enrolled in daily LAF-based programs demonstrated greater weight gain and a higher recovery rate than those in weekly programs [40].

Three studies found a higher recovery rate with LPF interventions than controls [29,30,34], and 3 studies reported greater weight gain [21,29,43]. However, LPFs were associated with lower weight gain in 3 studies [25,34,35] and lower recovery rate in 2 studies [25,38]. Two studies observed a shorter time to recovery with LPFs [21,22], although 1 study reported a longer time to recovery in the LPF group [21], and improvements in WHZ were reported in 3 studies [29,30,36]. Further details regarding individual study findings are provided in the Supplemental Table 2).

### Sensitivity analysis

We used a leave-one-out meta-analysis to evaluate how each study affected the overall pooled estimate. This was especially relevant because 2 studies employed distinct MAM classification criteria based on the NCHS standards [25,28]. By systematically excluding 1 study at a time and recalculating the pooled estimate, the results indicated variability in the pooled effect estimate, revealing the sensitivity of the meta-analysis to the exclusion of individual studies. The leave-one-out sensitivity analysis demonstrated that the pooled estimates for weight gain and recovery rate remained consistent after sequential exclusion of individual studies, indicating that the overall findings were robust and were not driven by any single study (Supplemental Figure 6).

### Publication bias

For the treatment of MAM using LAFs or LPFs compared with control interventions, the null hypothesis of funnel plot symmetry was not rejected across most outcomes, indicating no substantial publication bias. Specifically, for weight gain, Egger’s test showed nonsignificant results for both LAFs (*P* = 0.880) and LPFs (*P* = 0.476). Similarly, for recovery rate, no significant asymmetry was observed for LAFs (*P* = 0.384) or LPFs (*P* = 0.988). No publication bias was detected for height with LAFs (*P* = 0.974) or for MUAC gain with LPFs (*P* = 0.2061; LAFs, *P* = 0.084). However, marginal evidence of publication bias was observed for MUAC change in the LAFs group, WHZ in LAFs, and height in LPFs, where Egger’s test approached statistical significance (*P* ≈ 0.05). Although these borderline results may indicate potential small-study effects, it is important to note that Egger’s test may lack sufficient statistical power to detect bias when outcomes are informed by a small number of studies. Despite this, visual inspection of the funnel plots for these outcomes did not suggest substantial asymmetry. Comprehensive details of the publication bias assessment, including all funnel plots, are available in Supplemental Figures 7–11.

## Discussion

We reviewed and synthesized experimental evidence to determine the efficacy of local food interventions compared with SNFs for treating MAM in children. We tested the hypothesis that LAF and LPF interventions were non-inferior to SNFs for key outcomes such as recovery rate, weight gain, and time to recovery. These interventions used various locally sourced ingredients such as cereals, legumes, nuts, and seeds, with or without fortification with essential micronutrients. Our findings demonstrated that LAFs were inferior in recovery rate but non-inferior for weight gain. Time to recovery for LAF interventions was slightly longer than for SNFs but remained within the predefined non-inferiority margin. LPFs were non-inferior to SNFs across recovery and weight gain.

This analysis revealed a complex picture when comparing LAFs with SNFs. The inconsistency between recovery and weight gain can be attributed to 2 significant methodological considerations in the included studies. First, there was heterogeneity within the LAFs category; 3 of 4 studies evaluated nutritional counseling alone [29,33,38], with only 1 study providing food supplements made of local ingredients [22]. Nutritional counseling is an important behavioral strategy that encourages the consumption of home or local foods to support optimal child growth [44,45]. However, when implemented without direct food supplementation, its effectiveness may be influenced by household food availability, caregiver adherence, and the nutritional quality of accessible foods. Consequently, counseling alone may not consistently provide the energy density, protein quality, and micronutrient adequacy required for full recovery from MAM. In contrast, SNFs are formulated to deliver high-density and balanced energy in the form of macronutrients (protein, fat, and carbohydrates) and micronutrients [46]. Protein quality, derived particularly from animal sources such as milk, whey, or meat, is also critical, as it allows for high digestibility and essential amino acids [47]. These nutritional differences may partly explain why LAFs were associated with lower recovery rates despite remaining non-inferior for weight gain. Second, the studies included in the recovery and weight gain analyses did not always overlap, with only a few that measured both outcomes. Although the study populations were generally comparable, differences in outcome reporting and the studies contributing to each pooled estimate may have contributed to the divergent findings between recovery rate and weight gain.

Despite these limitations, LAFs can achieve short-term outcomes comparable with SNFs, such as weight gain and initial recovery, largely because caloric intake can be met through isocaloric LAF rations [12,48]. Nevertheless, LAFs often fall short in achieving adequate recovery rates, as successful rehabilitation requires more than energy provision alone. Optimal recovery depends on sufficient intake of bioavailable micronutrients to support metabolic and immune functions, high-quality protein to facilitate tissue repair, and safe foods free from contamination [49,50]. Additionally, SNFs are intended solely for the target child, whereas LAFs are often shared within the household, potentially reducing the amount of energy and nutrients available to the child and undermining the consistent, high-quality nutrient intake needed for complete and sustained recovery [51].

This study provides important evidence on the effectiveness of LPFs for the management of MAM. Overall, LPFs demonstrated outcomes comparable to SNFs for recovery rate, weight gain, and time to recovery, particularly when compared with FBF. When analyzed individually, LPFs were also non-inferior to FBF across these outcomes. Compared with RUSF, LPFs remained non-inferior for weight gain and time to recovery, whereas recovery outcomes were generally similar but did not strictly meet the predefined non-inferiority criterion. However, this comparison should be interpreted cautiously because it was based on a single study. The effectiveness of LPFs may partly reflect nutritional enhancement and processing methods intended to improve energy density, protein quality, micronutrient content, and nutrient bioavailability compared with unmodified locally sourced foods. Several LPF formulations included nutrient-enhancing ingredients or additives, including micronutrient premixes, although the composition and degree of fortification varied across studies. These formulation differences may have improved the nutritional value of the interventions and contributed to their comparability with SNFs. In addition, processing methods such as malting, germination, extrusion, fermentation, and roasting may improve nutrient quality by reducing antinutritional factors, including phytates and tannins, thereby enhancing protein digestibility and mineral absorption [[52], [53], [54], [55]].

These findings suggest that LPFs may serve as context-specific alternatives to SNFs for the management of MAM, particularly in resource-limited settings where dependence on imported products may create supply chain and sustainability challenges. Evidence from programmatic and implementation studies in countries such as Nigeria, Senegal, and Uganda has reported favorable recovery outcomes using locally formulated food-based interventions for MAM management [8]. In addition, the updated WHO guideline on wasting recognizes the role of nutrient-dense local foods for many children with MAM while reserving SNFs for children with higher-risk conditions [11]. However, global standards for the formulation, fortification, quality assurance, and therapeutic use of non-fortified local foods remain limited.

Beyond clinical outcomes, locally sourced food interventions may offer important programmatic and sustainability benefits. They can support local agriculture, strengthen food-processing capacity, create employment opportunities, improve community acceptance, and contribute to more sustainable nutrition programming [6,56]. Evidence from Mali and Sierra Leone also suggests that community-based MAM treatment models and supplementary food choices may be cost-effective, depending on the formulation and delivery approach [57,58]. Local production may reduce dependence on imported finished products and long-distance supply chains; however, potential environmental benefits should be interpreted cautiously because food system impacts depend on production methods, ingredients, processing, packaging, transport mode, and scale [[59], [60], [61]]. Successful implementation requires strong quality assurance, food safety monitoring, aflatoxin control, safe storage, and adequate water, sanitation, and hygiene infrastructure to ensure safe and consistent production. Therefore, LPF-based approaches could be considered for MAM management in settings with sufficient production capacity, regulatory oversight, and nutritional quality assurance systems.

Despite promising evidence, local food-based interventions for managing MAM face several critical implementation challenges, including the lack of global guidance and standards specific to locally produced foods, as well as food insecurity and seasonal fluctuations in food availability that can hinder implementation. Household food-sharing practices may also dilute the intended nutritional benefits for malnourished children, further complicating outcome assessment [8]. Financial sustainability poses another challenge, as securing consistent funding and maintaining quality standards for locally produced flours remains difficult. Finally, inadequate referral systems and weak integration with broader health services undermine the continuity of care and limit the overall effectiveness of these programs [62,63]. Thus, careful consideration of factors such as food-processing capacity, quality control to ensure nutritional adequacy, and the need for ongoing monitoring of program outcomes is required to maximize impact and sustainability [8]. In most LAF studies, nutritional counseling was used as the sole intervention for managing MAM, resulting in lower recovery rates, lower weight gain, and longer recovery times than SNFs. Therefore, it would be valuable to combine nutritional counseling with local food supplementation in a stepwise approach to evaluate optimized LAF formulations against SNFs.

This systematic review and meta-analysis has several limitations that should be considered when interpreting the findings. First, the number of studies included in pooled analyses for some key outcomes, particularly recovery rate for LPFs and time to recovery, was relatively small, which may have reduced statistical power and precision. Second, most included studies were conducted in SSA, which may limit generalizability to other regions with different dietary practices, health systems, socioeconomic contexts, disease burden, food security conditions, and baseline nutritional status. Third, several included studies had small sample sizes, which may have contributed to imprecise effect estimates.

In addition, the composition, preparation methods, and nutrient profiles of LAFs and LPFs varied substantially across studies, limiting comparability across interventions. The inclusion of nutrition-counseling-only interventions within the LAFs category may also have introduced conceptual and methodological heterogeneity because these interventions promoted the consumption and preparation of locally available household foods rather than providing standardized food supplementation or controlled nutrient compositions. Consequently, their effectiveness may have been influenced by factors such as household food availability, caregiver adherence, and the nutritional quality of accessible foods. However, sensitivity analyses, including leave-one-out analyses, demonstrated generally consistent findings and did not materially alter the overall interpretation of the results. Furthermore, the limited number of studies evaluating LAF interventions reduced the feasibility and interpretability of conducting separate pooled analyses for counseling-only interventions.

Minor modifications to the review framework were made after PROSPERO registration to improve alignment between the available evidence and the final analytical approach, particularly regarding intervention classification and the application of the non-inferiority framework. However, these changes did not affect the primary research question, target population, or overall study objectives. In addition, the absence of standardized global guidance for the formulation, fortification, quality assurance, and therapeutic use of LAFs and LPFs presents challenges for implementation, regulatory oversight, and scalability. These limitations highlight the need for more rigorous, geographically diverse, and methodologically harmonized research to strengthen the evidence base for locally sourced interventions in MAM management.

In conclusion, our meta-analyses demonstrated that LPFs are non-inferior to SNFs for recovery rate and weight gain in the management of MAM in children aged 6 to 59 mo, indicating that LPFs can be used as an alternative to SNFs, and their scale-up should be considered. Although LAFs showed lower overall recovery rates, they were comparable in promoting weight gain. This suggests that LAFs can support anthropometric improvements, but their effectiveness for full recovery is limited without fortification or enhanced nutrient density; however, additional studies of local food supplementation are needed to confirm this finding. Further research is needed to optimize the formulation and effectiveness of LAFs for children with MAM.

## Author contributions

The authors’ responsibilities were as follows – EAK, SHG, HYH, AMF, ECK, PB: contributed to the study design, including project conception, development of the overall research plan, and study oversight; EAK, GKP, HYH: conducted the literature search, study selection, data extraction, and risk-of-bias assessment; EAK, SHG, ECK, GKP, HYH: conducted the literature search, data extraction, and data acquisition; EAK, HYH: performed the statistical analyses and synthesized the data; EAK, HYH: drafted the manuscript; AMF, YDN, DN, ECK, TAS, KB, AK, RA-A, PB, SG, SHG: critically reviewed the manuscript and provided supervision; and all authors: read and approved the final manuscript.

## Data availability

The data and analytic code supporting the findings of this study will be made available upon reasonable request to the first author, Eskeziaw Abebe Kassahun (eskeziaw02@gmail.com), subject to applicable institutional and ethical approvals.

## Declaration of Generative AI and AI-assisted technologies in the writing process

During the preparation of this work, the author(s) used Rayyan, web-based AI tool inorder to support fulll-text review. After using this tool/service, the author(s) reviewed and edited the content, ectracted the necesary information and take full responsibility for the content of the publication.

## Funding

The authors reported no funding received for this study.

## Conflict of interest

The authors report no conflicts of interest.
