# Supplementary material for: Local Food Interventions for the Management of Moderate Acute Malnutrition (MAM) among Children Aged 6 to 59 Months: A Systematic Review and Non-inferiority Meta-Analysis
Source: Adv Nutr. 2026 Aug 7;17(9):100707. doi: 10.1016/j.advnut.2026.100707 (PMC13594452; doi:10.1016/j.advnut.2026.100707)
Supplement: Supplementary file 1 [file mmc1.docx]

**Local food interventions for the management of Moderate Acute Malnutrition (MAM) among children aged 6 to 59 months: A systematic review and non-inferiority meta-analysis**

Eskeziaw Abebe Kassahun^1^, Hamid Yimam Hassen^2,3^, Alinoor Mohamed Farah^1^, Yakob Desalegn Nigatu^1^, Debritu Nane^4,5^, Emily C. Keats^6^, Genet kiflemariam Paulos^1^, Tesfamichael Awoke Sissay^1^, Kemeria Barsenga^7^, Aweke kebede^7^, Robert Ackatia-Armah^7^, Paluku Bahwere^8^, Samson Gebremedhin^1^, Seifu Hagos Gebreyesus^1^*

^1^Department of Nutrition and Dietetics, School of Public Health, Addis Ababa University, College of Health Science, Addis Ababa University, Ethiopia

^2^Department of Family Medicine & Population Health, Faculty of Medicine and Health Sciences, University of Antwerp, Antwerp, Belgium

^3^Environmental Intelligence Unit, Flemish Institute for Technological Research (VITO), Boeretang 200, 2400, Mol, Belgium

^4^School of Public Health, College of Health Science and Medicine, Wolaita Sodo University, Ethiopia

^5^Institute for Global Nutrition, University of California Davis, USA

^6^Department of International Health, Johns Hopkins Bloomberg School of Public Health, Baltimore, USA

^7^World Food Program, Ethiopia

^8^Independent researcher

Eskeziaw Abebe Kassahun: Eskeziaw02@gmail.com

Hamid Yimam Hassen: Abdulhamidy71@gmail.com

Alinoor Mohammed Farah: alinuriana@yahoo.com

Yakob Desalegne Nigatu: yakobdesalegne@gmail.com

Debritu Nane: nanedebritu@gmail.com

Emily C. Keats: ekeats3@jh.edu

Genet kiflemariam Paulos: genetkiflemariam@gmail.com

Tesfamichael Awoke Sissay: tesjesus@gmail.com

Kemeria Barsenga: kemeria.barsenga@wfp.org

Aweke Kebede: aweke.kebede@wfp.org

Robert Ackatia-Armah: robert.ackatiaarmah@wfp.org

Paluku Bahwere: paluku.bahwere@gmail.com

Samson Gebremedhin: samsongmgs@yahoo.com

Seifu Hagos Gebreyesus: seif_h23@yahoo.com

*Corresponding author, Dr. Seifu Hagos Gebreyesus, Department of Nutrition and Dietetics, School of Public Health, Addis Ababa University, College of Health Science, Addis Ababa University, Ethiopia, email: seif_h23@yahoo.com, phone:+251911613577

**Source of funding:** This work received no funding

**Conflict of interest:** The authors declare no conflict of interest

Table of Contents

[**Appendix A:Supplementary data**	5](#_Toc230733267)

**Supplemental** [**Table 1. PRISMA 2020 checklist**	5](#_Toc230733268)

**Supplemental** [**Table 2: Search strategy for effectiveness of local food intervention for the management of MAM, 2025**	12](#_Toc230733269)

**Supplemental** [**Table 3: Characteristics of populations from the included trials in review**	20](#_Toc230733270)

**Supplemental** [**Table 4 (A): Cochrane risk of bias of included Randomized Controlled studies**	33](#_Toc230733271)

**Supplemental** [**Table 4 (B):** **Cochrane risk of bias of included Cluster-Randomized Controlled studies**	33](#_Toc230733272)

**Supplemental** [**Table 4 (C): Cochrane risk of bias of included non-Randomized Controlled studies**	34](#_Toc230733273)

**Supplemental** [**Table 5: Pooled effects of locally available foods (LAFs) and locally processed foods (LPFs) on secondary outcomes in the management of moderate acute malnutrition (MAM)**	35](#_Toc230733274)

**Supplemental** [**Figure 1. Forest plot for the mean difference in height gain between LAFs (A) and LPFs (B) compared to SNFs**	37](#_Toc230733275)

**Supplemental** [**Figure 2. Forest plot for the mean difference in MUAC gain between LAFs (A) and LPFs (B) compared to SNFs**	38](#_Toc230733276)

**Supplemental** [**Figure 3. Forest plot for the risk difference in deterioration rate LAFs (A) compared to SNFs**	39](#_Toc230733277)

**Supplemental** [**Figure 4. Forest plot for the risk difference death rate in LAFs (A) compared to SNFs**	39](#_Toc230733278)

**Supplemental** [**Figure 5. Forest plot for the mean difference in WHZ change at discharge for A) LAFs and B) LPFs compared to SNFs**	40](#_Toc230733279)

**Supplemental** [**Figure 8: Contour-enhanced funnel plots for Recovery rate in the LAFs (A) and LPFs (B) intervention groups.**	44](#_Toc230733280)

**Supplemental** [**Figure 9: Contour-enhanced funnel plots for height gain in the LAFs (A) and LPFs (B) intervention groups.**	45](#_Toc230733281)

**Supplemental** [**Figure 10: Contour-enhanced funnel plots for WHZ change in the LAFs intervention groups**	46](#_Toc230733282)

**Supplemental** [**Figure 11: Contour-enhanced funnel plots for MUAC in the LAFs (A) and LPFs (B) intervention groups.**	46](#_Toc230733283)

**Appendix A:****Supplementary data**

# Supplemental Table 1. PRISMA 2020 checklist

| **Section and Topic** | **Item #** | **Checklist item** | **Location where item is reported** |
| --- | --- | --- | --- |
| **TITLE** | | |  |
| Title | 1 | Identify the report as a systematic review. | 1 |
| **ABSTRACT** | | |  |
| Abstract | 2 | See the PRISMA 2020 for Abstracts checklist. | 5 |
| **INTRODUCTION** | | |  |
| Rationale | 3 | Describe the rationale for the review in the context of existing knowledge. | 5, 6 and 7 |
| Objectives | 4 | Provide an explicit statement of the objective(s) or question(s) the review addresses. | 7 and 8 |
| **METHODS** | | |  |
| Eligibility criteria | 5 | Specify the inclusion and exclusion criteria for the review and how studies were grouped for the syntheses. | 9, 10 and 11 |
| Information sources | 6 | Specify all databases, registers, websites, organisations, reference lists and other sources searched or consulted to identify studies. Specify the date when each source was last searched or consulted. | 8 |
| Search strategy | 7 | Present the full search strategies for all databases, registers and websites, including any filters and limits used. | 8 and supplement |
| Selection process | 8 | Specify the methods used to decide whether a study met the inclusion criteria of the review, including how many reviewers screened each record and each report retrieved, whether they worked independently, and if applicable, details of automation tools used in the process. | 9 |
| Data collection process | 9 | Specify the methods used to collect data from reports, including how many reviewers collected data from each report, whether they worked independently, any processes for obtaining or confirming data from study investigators, and if applicable, details of automation tools used in the process. | 8, 9 and 10 |
| Data items | 10a | List and define all outcomes for which data were sought. Specify whether all results that were compatible with each outcome domain in each study were sought (e.g. for all measures, time points, analyses), and if not, the methods used to decide which results to collect. | 10 and 11 |
|  | 10b | List and define all other variables for which data were sought (e.g. participant and intervention characteristics, funding sources). Describe any assumptions made about any missing or unclear information. | NA |
| Study risk of bias assessment | 11 | Specify the methods used to assess risk of bias in the included studies, including details of the tool(s) used, how many reviewers assessed each study and whether they worked independently, and if applicable, details of automation tools used in the process. | 12 |
| Effect measures | 12 | Specify for each outcome the effect measure(s) (e.g. risk ratio, mean difference) used in the synthesis or presentation of results. | 12, 13 and 14 |
| Synthesis methods | 13a | Describe the processes used to decide which studies were eligible for each synthesis (e.g. tabulating the study intervention characteristics and comparing against the planned groups for each synthesis (item #5)). | 13 |
|  | 13b | Describe any methods required to prepare the data for presentation or synthesis, such as handling of missing summary statistics, or data conversions. | 12, 13 and 14 |
|  | 13c | Describe any methods used to tabulate or visually display results of individual studies and syntheses. | 14 and 15 |
|  | 13d | Describe any methods used to synthesize results and provide a rationale for the choice(s). If meta-analysis was performed, describe the model(s), method(s) to identify the presence and extent of statistical heterogeneity, and software package(s) used. | 12 and 13 |
|  | 13e | Describe any methods used to explore possible causes of heterogeneity among study results (e.g. subgroup analysis, meta-regression). | 14 |
|  | 13f | Describe any sensitivity analyses conducted to assess robustness of the synthesized results. | 14 |
| Reporting bias assessment | 14 | Describe any methods used to assess risk of bias due to missing results in a synthesis (arising from reporting biases). | 13 and 14 |
| Certainty assessment | 15 | Describe any methods used to assess certainty (or confidence) in the body of evidence for an outcome. | 13 and 14 |
| **RESULTS** | | |  |
| Study selection | 16a | Describe the results of the search and selection process, from the number of records identified in the search to the number of studies included in the review, ideally using a flow diagram. | 15 |
|  | 16b | Cite studies that might appear to meet the inclusion criteria, but which were excluded, and explain why they were excluded. | 15 and16 |
| Study characteristics | 17 | Cite each included study and present its characteristics. | 16 and 17 |
| Risk of bias in studies | 18 | Present assessments of risk of bias for each included study. | 16 |
| Results of individual studies | 19 | For all outcomes, present, for each study: (a) summary statistics for each group (where appropriate) and (b) an effect estimate and its precision (e.g. confidence/credible interval), ideally using structured tables or plots. | 16, 17 and 18 |
| Results of syntheses | 20a | For each synthesis, briefly summarise the characteristics and risk of bias among contributing studies. | 17 and 18 |
|  | 20b | Present results of all statistical syntheses conducted. If meta-analysis was done, present for each the summary estimate and its precision (e.g. confidence/credible interval) and measures of statistical heterogeneity. If comparing groups, describe the direction of the effect. | 18, 19 and 20 |
|  | 20c | Present results of all investigations of possible causes of heterogeneity among study results. | 219, 20and 21 |
|  | 20d | Present results of all sensitivity analyses conducted to assess the robustness of the synthesized results. | 21 |
| Reporting biases | 21 | Present assessments of risk of bias due to missing results (arising from reporting biases) for each synthesis assessed. | 23 and 24 |
| Certainty of evidence | 22 | Present assessments of certainty (or confidence) in the body of evidence for each outcome assessed. | 17, 18, 19, 20 and 22 |
| **DISCUSSION** | | |  |
| Discussion | 23a | Provide a general interpretation of the results in the context of other evidence. | 22,23 and 24 |
|  | 23b | Discuss any limitations of the evidence included in the review. | 24,25, 26 and 27 |
|  | 23c | Discuss any limitations of the review processes used. | 25, 26 and 27 |
|  | 23d | Discuss implications of the results for practice, policy, and future research. | 27 |
| **OTHER INFORMATION** | | |  |
| Registration and protocol | 24a | Provide registration information for the review, including register name and registration number, or state that the review was not registered. | 5 and 8 |
|  | 24b | Indicate where the review protocol can be accessed, or state that a protocol was not prepared. | NA |
|  | 24c | Describe and explain any amendments to information provided at registration or in the protocol. | NA |
| Support | 25 | Describe sources of financial or non-financial support for the review, and the role of the funders or sponsors in the review. | 28 |
| Competing interests | 26 | Declare any competing interests of review authors. | 28 |
| Availability of data, code and other materials | 27 | Report which of the following are publicly available and where they can be found: template data collection forms; data extracted from included studies; data used for all analyses; analytic code; any other materials used in the review. | 28 |

From: Page MJ, McKenzie JE, Bossuyt PM, Boutron I, Hoffmann TC, Mulrow CD, et al. The PRISMA 2020 statement: an updated guideline for reporting systematic reviews. BMJ 2021;372:n71. doi: 10.1136/bmj.n71

# **Supplemental Table 2: Search strategy for effectiveness of local food intervention for the management of MAM, 2025**

**Box S2: Strategy in Medline**

| **Search no** | **search Query** |
| --- | --- |
| **#4** | **#1 AND #2 AND #3** |
| #3 | "local food"[tiab:~3] OR "Local food*"[Title/Abstract] OR "Locally available food*"[Title/Abstract] OR "Food intervention"[Title/Abstract] OR "Fortified blended food"[Title/Abstract] OR voucher* [Title/Abstract] OR "Traditional food*"[Title/Abstract] OR "Home food*"[Title/Abstract] OR "Community level intervention*"[Title/Abstract] OR "Local ingredients-based supplement*"[Title/Abstract] OR "Ready-to-use supplementary food"[Title/Abstract] OR "Ready-to-use therapeutic food"[Title/Abstract] OR "Specially formulated food*"[Title/Abstract] OR "Supplementary food*"[Title/Abstract] OR "Locally produced food*"[Title/Abstract] OR "corn soy blend"[Title/Abstract] OR "corn soya blend"[Title/Abstract] OR "CSB"[Title/Abstract] OR "locally prepared therapeutic diet"[Title/Abstract] OR "home made food*"[Title/Abstract] OR "Homemade food*"[Title/Abstract] OR "home-made food*"[Title/Abstract] OR "home made formula"[Title/Abstract] OR "Homemade formula"[Title/Abstract] OR "home-made formula"[Title/Abstract] OR "cash*"[Title/Abstract] OR "financial support"[Title/Abstract] OR "conditional cash transfer"[Title/Abstract] OR "unconditional cash transfer"[Title/Abstract] OR "income supplement"[Title/Abstract] OR "support grant"[Title/Abstract] |
| #2 | "undernutrition"[Title/Abstract] OR "under-nutrition"[Title/Abstract] OR "under nutrition"[Title/Abstract] OR "undernourish*"[Title/Abstract] OR "under nourish*"[Title/Abstract] OR "underfed"[Title/Abstract] OR "malnutrition"[Title/Abstract] OR "mal-nutrition"[Title/Abstract] OR "malnourish*"[Title/Abstract] OR "mal nourish*"[Title/Abstract] OR "deficiency disease"[Title/Abstract] OR "deficiency diseases"[Title/Abstract] OR "nutrition deficiency"[Title/Abstract] OR "nutritional deficiency"[Title/Abstract] OR "nutrition disease"[Title/Abstract] OR "nutrition diseases"[Title/Abstract] OR "nutritional disease"[Title/Abstract] OR "nutritional diseases"[Title/Abstract] OR "nutrition disorder"[Title/Abstract] OR "nutrition disorders"[Title/Abstract] OR "nutritional disorder"[Title/Abstract] OR "nutritional disorders"[Title/Abstract] OR "nutrient deficiency"[Title/Abstract] OR "nutrient disease"[Title/Abstract] OR "marasmus"[Title/Abstract] OR "kwashiorkor"[Title/Abstract] OR "emaciat*"[Title/Abstract] OR "moderate acute malnutrition"[Title/Abstract] OR "MAM"[Title/Abstract] OR "wasted"[Title/Abstract] OR "wasting"[Title/Abstract] OR "failure to thrive"[Title/Abstract] OR "growth disorders"[Title/Abstract] OR "starvation"[Title/Abstract] OR "starving"[Title/Abstract] OR "underweight"[Title/Abstract] OR "under-weight"[Title/Abstract] OR "under-weight"[Title/Abstract] OR "thinness"[Title/Abstract] OR "leanness"[Title/Abstract] OR "arm-circumference"[Title/Abstract] OR "arm-circumference"[Title/Abstract] OR "weight-for-age"[Title/Abstract] OR "weight-for-age"[Title/Abstract] OR "weight-for-height"[Title/Abstract] OR "weight-for-height"[Title/Abstract] OR "anthropometr*"[Title/Abstract] |
| #1 | "child*"[Title/Abstract] OR "infant*"[Title/Abstract] OR "toddler"[Title/Abstract] OR "preschool" OR "baby"[Title/Abstract] OR "babies"[Title/Abstract] OR "under five"[Title/Abstract] OR "under-five"[Title/Abstract] OR "under-5"[Title/Abstract] OR "under 5"[Title/Abstract] |

**Box S2- Search strategy in Web of science**

| #1 | TS=(child* OR infant* OR toddler OR preschool OR baby OR babies OR "under five" OR under-five OR under-5 OR "under 5") and Pediatric (OR – Search within topic) |
| --- | --- |
| #2 | TS=(undernutrition OR under-nutrition OR "under nutrition" OR undernourish* OR "under nourish*" OR underfed OR malnutrition OR mal-nutrition OR malnourish* OR "mal nourish*" OR "deficiency disease" OR "deficiency diseases" OR "nutrition deficiency" OR "nutritional deficiency" OR "nutrition disease" OR "nutrition diseases" OR "nutritional disease" OR "nutritional diseases" OR "nutrition disorder" OR "nutrition disorders" OR "nutritional disorder" OR "nutritional disorders" OR "nutrient deficiency" OR "nutrient disease" OR marasmus OR kwashiorkor OR emaciat* OR "moderate acute malnutrition" OR MAM OR wasted OR wasting OR "failure to thrive" OR "growth disorders" OR starvation OR starving OR underweight OR under-weight OR under-weight OR thinness OR leanness OR arm-circumference OR arm-circumference OR weight-for-age OR weight-for-age OR weight-for-height OR weight-for-height OR anthropometr*) |
| #3 | TS=(local NEAR/3 food OR "Local food*" OR "Locally available food*" OR "Food intervention" OR "Fortified blended food" OR voucher* OR "Traditional food*" OR "Home food*" OR "Community level intervention*" OR "Local ingredients-based supplement*" OR "Ready-to-use supplementary food" OR "Ready-to-use therapeutic food" OR "Specially formulated food*" OR "Supplementary food*" OR "Locally produced food*" OR "corn soy blend" OR "corn soya blend" OR CSB OR "locally prepared therapeutic diet" OR "home made food*" OR "Homemade food*" OR "home-made food*" OR "home made formula" OR "Homemade formula" OR "home-made formula" OR cash* OR "financial support" OR "income supplement" OR "support grant") |
| **#4** | **#1 AND #2 AND #3** |

**Box S2: Search strategy in Embase**

| **#1** | ((local NEAR/3 food):ti,ab,kw) OR 'local food*':ti,ab,kw OR 'locally available food*':ti,ab,kw OR 'food intervention':ti,ab,kw OR 'fortified blended food':ti,ab,kw OR voucher*:ti,ab,kw OR 'traditional food*':ti,ab,kw OR 'home food*':ti,ab,kw OR 'community level intervention*':ti,ab,kw OR 'local ingredients-based supplement*':ti,ab,kw OR 'ready-to-use supplementary food':ti,ab,kw OR 'ready-to-use therapeutic food':ti,ab,kw OR 'specially formulated food*':ti,ab,kw OR 'supplementary food*':ti,ab,kw OR 'locally produced food*':ti,ab,kw OR 'corn soy blend':ti,ab,kw OR 'corn soya blend':ti,ab,kw OR csb:ti,ab,kw OR 'locally prepared therapeutic diet':ti,ab,kw OR 'home made food*':ti,ab,kw OR 'homemade food*':ti,ab,kw OR 'home-made food*':ti,ab,kw OR 'home made formula':ti,ab,kw OR 'homemade formula':ti,ab,kw OR 'home-made formula':ti,ab,kw OR cash*:ti,ab,kw OR 'financial support':ti,ab,kw OR 'income supplement':ti,ab,kw OR 'support grant':ti,ab,kw |
| --- | --- |
| **#2** | child*:ti,ab OR infant*:ti,ab OR toddler:ti,ab OR preschool OR baby:ti,ab OR babies:ti,ab OR 'under five':ti,ab OR 'under 5':ti,a |
| **#3** | undernutrition:ti,ab OR 'under nutrition':ti,ab OR undernourish*:ti,ab OR 'under nourish*':ti,ab OR underfed:ti,ab OR malnutrition:ti,ab OR 'mal nutrition':ti,ab OR malnourish*:ti,ab OR 'mal nourish*':ti,ab OR 'deficiency disease':ti,ab OR 'deficiency diseases':ti,ab OR 'nutrition deficiency':ti,ab OR 'nutritional deficiency':ti,ab OR 'nutrition disease':ti,ab OR 'nutrition diseases':ti,ab OR 'nutritional disease':ti,ab OR 'nutritional diseases':ti,ab OR 'nutrition disorder':ti,ab OR 'nutrition disorders':ti,ab OR 'nutritional disorder':ti,ab OR 'nutritional disorders':ti,ab OR 'nutrient deficiency':ti,ab OR 'nutrient disease':ti,ab OR marasmus:ti,ab OR kwashiorkor:ti,ab OR emaciat*:ti,ab OR 'moderate acute malnutrition':ti,ab OR mam:ti,ab OR wasted:ti,ab OR wasting:ti,ab OR 'failure to thrive':ti,ab OR 'growth disorders':ti,ab OR starvation:ti,ab OR starving:ti,ab OR underweight:ti,ab OR 'under weight':ti,ab OR thinness:ti,ab OR leanness:ti,ab OR 'arm circumference':ti,ab OR 'weight for age':ti,ab OR 'weight for height':ti,ab OR anthropometr*:ti,ab |
| **#4** | **(#1 AND #2 AND #3)** |

**Box S2: Search strategy in Cochrane**

| #1 | child*:ti,ab OR infant*:ti,ab OR toddler:ti,ab OR preschool OR baby:ti,ab OR babies:ti,ab OR "under five":ti,ab OR under-five:ti,ab OR under-5:ti,ab OR "under 5":ti,ab |
| --- | --- |
| #2 | undernutrition:ti,ab OR under-nutrition:ti,ab OR "under nutrition":ti,ab OR undernourish*:ti,ab OR ("under" NEXT nourish*):ti,ab OR underfed:ti,ab OR malnutrition:ti,ab OR mal-nutrition:ti,ab OR malnourish*:ti,ab OR ("mal" NEXT nourish*):ti,ab OR "deficiency disease":ti,ab OR "deficiency diseases":ti,ab OR "nutrition deficiency":ti,ab OR "nutritional deficiency":ti,ab OR "nutrition disease":ti,ab OR "nutrition diseases":ti,ab OR "nutritional disease":ti,ab OR "nutritional diseases":ti,ab OR "nutrition disorder":ti,ab OR "nutrition disorders":ti,ab OR "nutritional disorder":ti,ab OR "nutritional disorders":ti,ab OR "nutrient deficiency":ti,ab OR "nutrient disease":ti,ab OR marasmus:ti,ab OR kwashiorkor:ti,ab OR emaciat*:ti,ab OR "moderate acute malnutrition":ti,ab OR MAM:ti,ab OR wasted:ti,ab OR wasting:ti,ab OR "failure to thrive":ti,ab OR "growth disorders":ti,ab OR starvation:ti,ab OR starving:ti,ab OR underweight:ti,ab OR under-weight:ti,ab OR under-weight:ti,ab OR thinness:ti,ab OR leanness:ti,ab OR arm-circumference:ti,ab OR arm-circumference:ti,ab OR weight-for-age:ti,ab OR weight-for-age:ti,ab OR weight-for-height:ti,ab OR weight-for-height:ti,ab OR anthropometr*:ti,ab |
| #3 | local food[tiab:~3] OR ("Local" NEXT food*):ti,ab OR ("Locally available" NEXT food*):ti,ab OR "Food intervention":ti,ab OR "Fortified blended food":ti,ab OR voucher*:ti,ab OR ("Traditional" NEXT food*):ti,ab OR ("Home" NEXT food*):ti,ab OR ("Community level" NEXT intervention*):ti,ab OR ("Local ingredients-based" NEXT supplement*):ti,ab OR "Ready-to-use supplementary food":ti,ab OR "Ready-to-use therapeutic food":ti,ab OR ("Specially formulated" NEXT food*):ti,ab OR ("Supplementary" NEXT food*):ti,ab OR ("Locally produced" NEXT food*):ti,ab OR "corn soy blend":ti,ab OR "corn soya blend":ti,ab OR CSB:ti,ab OR "locally prepared therapeutic diet":ti,ab OR ("home made" NEXT food*):ti,ab OR ("Homemade" NEXT food*):ti,ab OR ("home-made" NEXT food*):ti,ab OR "home made formula":ti,ab OR "Homemade formula":ti,ab OR "home-made formula":ti,ab OR cash*:ti,ab OR "financial support":ti,ab OR "conditional cash transfer":ti,ab OR "unconditional cash transfer":ti,ab OR "income supplement":ti,ab OR "support grant":ti,ab |
| **#4** | **#1 AND #2 AND #3** |

**Box S5: Search strategy in ProQuest**

| #1 | TI,AB[67] OR TI,AB[67] OR TI,AB(toddler) OR preschool OR TI,AB(baby) OR TI,AB(babies) OR TI,AB("under five") OR TI,AB(under-five) OR TI,AB(under-5) OR TI,AB("under 5") |
| --- | --- |
| #2 | TI,AB(undernutrition) OR TI,AB(under-nutrition) OR TI,AB("under nutrition") OR TI,AB(undernourish*) OR TI,AB("under nourish*") OR TI,AB(underfed) OR TI,AB(malnutrition) OR TI,AB(mal-nutrition) OR TI,AB(malnourish*) OR TI,AB("mal nourish*") OR TI,AB("deficiency disease") OR TI,AB("deficiency diseases") OR TI,AB("nutrition deficiency") OR TI,AB("nutritional deficiency") OR TI,AB("nutrition disease") OR TI,AB("nutrition diseases") OR TI,AB("nutritional disease") OR TI,AB("nutritional diseases") OR TI,AB("nutrition disorder") OR TI,AB("nutrition disorders") OR TI,AB("nutritional disorder") OR TI,AB("nutritional disorders") OR TI,AB("nutrient deficiency") OR TI,AB("nutrient disease") OR TI,AB(marasmus) OR TI,AB(kwashiorkor) OR TI,AB(emaciat*) OR TI,AB("moderate acute malnutrition") OR TI,AB(MAM) OR TI,AB(wasted) OR TI,AB(wasting) OR TI,AB("failure to thrive") OR TI,AB("growth disorders") OR TI,AB(starvation) OR TI,AB(starving) OR TI,AB(underweight) OR TI,AB(under-weight) OR TI,AB(under-weight) OR TI,AB(thinness) OR TI,AB(leanness) OR TI,AB(arm-circumference) OR TI,AB(arm-circumference) OR TI,AB(weight-for-age) OR TI,AB(weight-for-age) OR TI,AB(weight-for-height) OR TI,AB(weight-for-height) OR TI,AB(anthropometr*) |
| #3 | local food[tiab:~3] OR TI,AB("Local food*") OR TI,AB("Locally available food*") OR TI,AB("Food intervention") OR TI,AB("Fortified blended food") OR TI,AB(voucher*) OR TI,AB("Traditional food*") OR TI,AB("Home food*") OR TI,AB("Community level intervention*") OR TI,AB("Local ingredients-based supplement*") OR TI,AB("Ready-to-use supplementary food") OR TI,AB("Ready-to-use therapeutic food") OR TI,AB("Specially formulated food*") OR TI,AB("Supplementary food*") OR TI,AB("Locally produced food*") OR TI,AB("corn soy blend") OR TI,AB("corn soya blend") OR TI,AB(CSB) OR TI,AB("locally prepared therapeutic diet") OR TI,AB("home made food*") OR TI,AB("Homemade food*") OR TI,AB("home-made food*") OR TI,AB("home made formula") OR TI,AB("Homemade formula") OR TI,AB("home-made formula") OR TI,AB(cash*) OR TI,AB("financial support") OR TI,AB("conditional cash transfer") OR TI,AB("unconditional cash transfer") OR TI,AB("income supplement") OR TI,AB("support grant") |
| **#4** | **(#1 AND #2 AND #3)** |

# **Supplemental Table 3: Characteristics of populations from the included trials in review**

| **no** | **Author, Year** | **Study Design and Setting** | **Participants and Admission criteria** | **Intervention/Control and Duration** | **Primary outcomes** | **Main findings** | **Comparison status** |
| --- | --- | --- | --- | --- | --- | --- | --- |
| 1 | Ackatia-Armah et al., 2015 | cRCT rural setting in Diola Health District, Bamako, Mali | 1264 children aged 6–35 months **Admission criteria:** -WHZ <-2 and ≥-3 (WHO Growth Standards) or MUAC <12.5 cm and ≥11.5 cm or WHZ <80% and ≥70% of the NCHS median of MUAC <12.0 and >11.0 cm | **Group 1:** 500 kcal/day of fortified flour -Misola (n = 306) **(LPF)** **Group 2:** 500 kcal/day of locally milled flours plus oil, sugar, and micronutrient powder (LMF) (n=281)(**LPF**)  **Group 3:** 500 kcal/day of corn–soy blend (**CSB++**), **FBF** n = 342) **Group 4:** 500 kcal/day of ready-to-use supplementary food (**RUSF**) (n = 335) **Duration:** 12 weeks | MUAC gain  Weight gain  Height gain  Recovery rate  WHZ | - lipid-based supplementary food (RUSF) had greater weight gains and recovery rates than those treated with locally produced Misola - For length change, RUSF and CSB++ differed significantly from LMF. Sustained recovery rates were higher with RUSF than with Misola and LMF | **Comparisons**  LPF vs FBF  LPF vs RUSF |
| 2 | McDonald et al., 2019 | cRCT  20 health centers in the health district of Dioila, Mali.  Sub sample study of Ackatia-Armah | 286 children aged 6–35 month  **Admission criteria:**  -WHZ <-2 and ≥-3 (WHO Growth Standards) or MUAC <12.5 cm and ≥11.5 cm or WHZ <80% and ≥70% of the NCHS median | **Group 1**: 500 kcal/d fortified flour-Misola, **LPF**(N=82)  **Group 2**: 500 kcal/d a less-refined cereal-legume milled flour mix (LMF) (**LPF**) (n=55)  **Group 3**: 500 kcal/d Corn-Soy Blend "Plus Plus" (**CSB++**) (FBF) (n=85)  **Group 4:** 500 kcal/d a lipid-based RUSF (**RUSF**) (n=64)  **Duration:** 12 weeks | Weight gain  WLZ  Fat mass  Fat mass index  Recovery | Children receiving RUSF showed greater gains in weight, fat mass, fat mass index, and weight-for-length z score compared to those receiving locally processed, fortified flour  Recovery from MAM was associated with significant increases in fat mass and other body composition parameters | Sub-sample for Ackatia-Armah et al., 2015 and will consider in systematic review only |
| 3 | Kajjura et al, 2019 | cRCT 24 rural parishes Arua district, North Western Uganda | 220 mother–child pairs aged 6–18 months **Admission criteria:** WHZ <-2 and ≥-3 | **Group 1:** 675 kcal/day of an active malt, extruded maize and soy sorghum-based porridge(MSBP), **LPF**(n=104) **Group 2:** 600 kcal/day of CSB Plus (**FBF**) (N= 110) **Duration:** 3 months | Weight gain  Length/height  WHZ  Recovery rate | - No difference in weight and length gain - Difference in mean weight-for-age Z-scores in the treatment group improved compared to control | **Comparison**  LPF vs FBF |
| 4 | Javan et al., 2017 | RCT  Heshmatiyeh Hospital in Sabzevar city in Iran | 64 infants, aged 9 to 24 months  **Admission criteria:** WLZ or WHZ < -2 and ≥ -3 | **Group 1**: 273 kcal/day Shadameen + vitamin & mineral **LPF (n=35)**  **Group 2**: vitamin+mineral+ nutrional counseling counseling) **LAF** (n=33)  **Duration:** 3 months | Weight  WHZ  LAZ  Length  Recovery rate | - Weight and WLZ and WAZ were greater compared other group - The recovery rate in the food supplemented group was significantly higher than the other group - There were no significant differences in LAZ at the end of the study between the two groups | **Comparison**  LAF vs LPF |
| 5 | Mostafa et al.,2024 | RCT  Mirpur slum of Dhaka, Bangladesh | 124 children aged 12-18 month  **Admission criteria:** WLZ or WHZ < -2 and ≥ -3 | **Group 1**: Locally produced microbiota-directed complementary food (MDCF-2) from **LPF (n=59)**  **Group 2:** standard ready-to-use supplementary food (RUSF), **RUSF (n=59)**  **Duration: 3 months** | Weight  Height  MUAC  length | - A statistically significant decrease in the rate of LAZ decline in children that had received 3 months of MDCF-2 - Higher MUAC, weight and length gain in MDCF-2 group | Comparison  LPF vs RUSF |
| 6 | Chen et al., 2021 | RCT  Mirpur slum of Dhaka, Bangladesh | 1118 children aged 12-18 month  **Admission criteria:** WLZ or WHZ < -2 and ≥ -3 | **Group 1**: Locally produced microbiota-directed complementary food (MDCF-2) from **LPF (n=59)**  **Group 2:** standard ready-to-use supplementary food (RUSF), **RUSF (n=59)**  **Duration Every week changes** | Weight  Height  MUAC | - The rates of change in the weight-for-length and weight-for-age z scores are consistent with a benefit of MDCF-2 on growth over the course of the study, including the 1-month follow-up | It is part of Mostafa et al. (2024) and assessed weekly changes |
| 7 | Costa et al., 2020 | RCT  Imperatriz, Brazil | 30 children aged between 2 and  5 years  **Admission criteria:** WHZ <-2 and ≥-3 (WHO Growth Standards) | **Group 1**: Two daily tablespoons (12 g) cashew nut flour, **LAF (n=15)**  **Group 2:** usual healthcare, **Standard care, no intervention [1] (n=15)**  **Duration:** 24 week | Weight,  Height,  BMI) | - Weight and height were consistently higher in the cashew nut flour group compared to the control group, although the differences were not statistically significant | Will be included in systematic review only  comparison  LAF vs LAF |
| 8 | Makori, N.,et al 2024 | RCT  Chamwino and Ikungi Districts in Central Tanzania. | 480 Children aged 6 to 59 months  **Admission criteria:** WHZ < -2 or MUAC < 12.5 cm | **Group 1**: 100 g/day of locally produced RUF + IYCF counseling, **LAF (n=181)**  **Group 2**: 100 g of CSB+/day + IYCF counseling, **FBF (n=165)**  **Group 3**: IYCF counseling only, **Nutritional counseling, LAF** (n=66)  **Duration**: 3 months | Recovery rate, weight gain, height gain  Deterioration rate  MUAC gain | - RUSF resulted in slightly greater weight and MUAC gains compared to CSB+ - CSB+ led to slightly greater height gains than RUSF - No significant differences were observed between CSB+ and RUSF in terms of recovery rate, weight gain, and MUAC gain | Comparison  LAF vs FBF |
| 9 | Nikièma et al., 2015 | RCT  health district of Hounde´, Western region of Burkina Faso | 1824 children aged 6–24 month  **Admission criteria:** WHZ <-2 and ≥-3 | **Group 1:** Daily 50 g fortified spread (locally produced soy-based RUSF), **LPF (n=694)**  **Group 2:** Daily 65 g CSB++, FBF **(n=675)**  **Group 3:** child-centered communication and nutrition counseling to caretakers, **Nutritional counseling, LAF (n=605)**  **Duration**: 3 months | Recovery rate, WHZ, HAZ, Weight gain, Length gain, MUAC gain | - Recovery rate was significantly lower with CCC (57.8%) than with CSB++ (74.5%) and RUSF (74.2%) - CSB+ showed slightly higher recovery rate than RUSF - Mothers’ attendance at health facilities was also substantially lower in the CCC arm | **Comparison**  LAF vs FBF  LPF vs FBF  LAF vs LPF |
| 10 | Nane et al, 2021 | RCT Damot Pulassa district, Wolaita, Southern Ethiopia | 324 children aged 6–59 months **Admission criteria:** WHZ <-2 and ≥-3 (WHO Growth Standards) or MUAC <12.5 cm and ≥11.5 cm | **Group 1:** 699 kcal/day of Local ingredients based supplement (LIBS) **LAF (n=162)** **Group 2:** 751 kcal/day of CSB+, **FBF (n=162)** **Duration:** 12 weeks | Recovery rate, time to recovery, weight gain, , MUAC gain, and height gain | - LIBS showed higher recovery rate, weight gain, MUAC gain, height gain and short recovery time than CSB+ | **Comparison**  LAF vs FBF |
| 11 | LaGrone N, et al., 2012 | RCT  Rural setting in South TFC, Malawi | 2712 children aged 6–59 month  **Admission criteria:** WHZ <-2 and ≥-3 (WHO Growth Standards) | **Group 1**:Children received 75 kcal CSB++ / kg/day, **FBF** (n=888)  **Group 2**: locally produced soy RUSF, Locally produced **RUSF** (n=906)  **Group 3**: imported soy/whey RUSF , **RUSF (n=918)**  **Duration**: 12 week. | Time to recovery,  Recovery rate weight gain, length gain,  MUAC gain, Deterioration rate | - CSB++ had similar recovery rates to both RUSF, but slower weight gain and a 2-day longer recovery time | **Comparison**  Locally FBF vs standard RUSF |
| 12 | Thakwalakwa,C., et al,2010 | RCT  Lungwena, Mangochi district of Malawi.. | 188 children aged 6-15 month  **Admission criteria:** WAZ <-2 ,  (NCHS/CDC) growth reference | **Group 1:** 500 g of locally produced CSB weekly **FBF (n=45)**  **Group 2:** 300 g of Lipid-based nutrient supplements (LNS) weekly **RUSF (N=47)**  **Group 3:** did not receive any food supplement, **Standard care, LAF (58)**  **Duration**: 12 week | Weight change, mean length changes,  WLZ,  LAZ,  MUAC | - There was no difference in overall body weight increases, the lipid-based supplement group showed greater weight gain and improved weight-for-age Z-score compared to the control - Changes in weight and weight-for-age Z-score did not differ between the corn-soy blend and control groups | **Comparison**  LAF vs FBF  LAF vs RUSF  FBF vs RUSF |
| 13 | Amegovu AK. et al. 2014 | cRCT Two health centers Kakamongole and Namalu in Nakapiripirit district, Karamoja, Uganda | 440 children aged 6–59 months **Admission criteria:** -WHZ <-2 and ≥-3 (WHO Growth Standards) | **Group 1:** 1200 kcal/day of a Sorghum Peanut Blend (SPB) (**LPF**) (n=194) **Group 2:** 1228 kcal per day of Corn soy blend plus (CSB+) (FBF) (n=198) **Duration:** 3 months | Recovery rate  WAZ  HAZ | - There was no significant difference in overall recovery rates between the Shadameen group (68.4%) and the control group (31.6%). - The Shadameen group showed greater rates of weight gain, and WLZ and WAZ increase in comparison with the control group | **Comparison**  LPF vs FBF |
| 14 | Matilsky, et al. 2009 | RCT Rural setting in the southern region of Malawi | 1362 children aged 6–60 months **Admission criteria:** WHZ <-2 and ≥-3 | **Group 1:** 75 kcal/kg/day of a milk/peanut fortified spread (**LNS**), **RUSF** (n = 465)  **Group 2:** 75 kcal/kg/day of a soy/peanut fortified spread (**LNS**), RUSF (n = 450) **Group 3:** 75 kcal/kg/day of a corn–soy blend **(FBF)(**n = 447) **Duration:** 8 weeks | Recovery rate  Develop oedema  Death  WHZ at discharge  HAZ at discharge | - Children receiving FS, whether milk/peanut or soy/peanut based, showed higher recovery rates and greater weight gain compared to those receiving CSB. - Soy/peanut FS is a cost-effective alternative to milk/peanut FS with similar recovery rates and nutritional benefits | **Comparison**  RUSFFs vs FBF |
| 15 | Roy et al., 2005 | Shahrasti, a sub-district in Chandpur district in Bangladesh | 282 MAM children 6 to 24 months  Admission criteria: weight-for-age between 61-75% of median of the NCHS | **Group 1:** Intensive nutrition education (LAF(n=93)  Group 2 (INE+SF): Intensive nutrition education plus supplementary feeding (300 kcal/day, 8–9 g protein) using a khichuri made from local ingredients (rice, pulse, molasses, oil), LAF (n=99)  Group 3 (BINP): Fortnightly nutrition education from BINP community nutrition promoters (standard care) (LAF n=90).  Duration: 3 months. | Recovery rate  Weight gain  WHZ  WAZ | - Recovery from wasting was highest in the INE + SF group (47%), followed by INE (37%) and control (18%). At 6-month follow-up, rates rose to 86% (INE + SF), 59% (INE), and 30% (control) | **Comparison**  LAF vs LAF  . |
| 16 | Fauveau et al., 1992 | Bangladesh | 134 MAM children Aged 6 to 12 months,  Admission criteria: MUAC > 110 and <129 mm, living in bamboo structure | **Group 1**: mothers received, weekly, 450 g of pre-mixed roasted and packed rice, wheat and lentil powder along with 90 g of cooking oil: 163 kcal/100 g energy (**LAF**, n=48)  **Group 2:** nutrition education (**LAF**, N=43) | Weight gain | - Food group have larger weight gain in first 3 months but not whole 6 months | **Comparison**  LAF vs LAF |
| 17 | Kohlmann et al., 2019 | Rural Ghana | 689 MAM aged 6 to 59 months  Admission criteria: MUAC≥11.5 cm,<12.5 cm WLZ between -2 and -3 SD | **Group 1: 75 kcal/kg/day standard RUTF-**contained peanut paste, sugar, nonfat dried milk, vegetable oil, a premix containing concentrated minerals and vitamins, and an emulsifier  (**RUSF**, n=426)  **Group** **2**: **75 kcal/kg/day** locally produced alternative RUTF: locally available soybean and sorghum flour, canola oil, sugar, a vi-tamin and mineral premix, as well as less nonnu-tritive emulsifier(**RUSF**, n=443)  Duration: 3 months | Recovery rate  Death  Weight gain  MUAC gain | - Among children with moderate wasting, recovery rates were lower for alternative RUTF, 386 of 443 (87%), than standard RUTF, 397 of 426 (93%) | **Comparation**  RUSTF vs RUSF |
| 18 | Nackers et al., 2010 | Magaria department, Zinder region, South of Niger | 1737 MAM Cchildren WHM F% from 70 to <80% (NCHS reference), without oedema and with a mid-upper arm circumference (MUAC) 110 mm | **Group 1: RURF(n=215)** RUTF [Plumpy’Nut (Nutriset, Malaunay, France) two packs daily, i.e. 1000 kcal day1 ]  **Group 2: CSB (n=236),** a CSB pre-mix (1750 g of CSB, 175 ml of vegetable oil and 105 g of sugar, i.e. 1231 kcal day1 ) |  | - RUTF was significantly more effective than corn/soy-blend (CSB) pre-mix in treating MAM among children in Niger, leading to higher recovery rates, faster weight gain, shorter treatment duration, and fewer transfers to inpatient care. | **Comparison**  FBF vs RUSF |
| 19 | Stobaugh et al., 2016 | Malawi’s | Children aged 6–59 mo with MAM,  Admission criteria: MUAC of 11.5–12.4 cm without bipedal edem | **Group 1:Soy RUSF (n = 1086)**  **Group 2: Whey RUSF (n = 1144)** |  | - A novel whey-based RUSF led to significantly higher recovery rates and improved growth outcomes in children with moderate acute malnutrition compared to a soy-based RUSF, despite providing less total protein and energy | **Comparison**  RUSF vs RUSF |
| 20 | Purwestri et al., 2013 | Indonesia, | 54 children aged 6-60 months  **Admission criteria:** WHZ <-2 and ≥-3 (WHO Growth Standards) | **Group 1:** Daily locally produced Ready-to-Use Food (RUF) biscuits, **locally RUSF (n=34)**  **Group 2:** Weekly Locally Produced RUF-Nias Biscuits (LPF)(**n=20**)  **Duration:** 62 days | Length of stay, weight gain, height gain, WHZ, MUAC | - Children in the daily program showed greater weight gain compared to those in the weekly program - A higher proportion of children in the daily program reached the target compared to the weekly program | Included in systematic review only  RUSF compared in daily vs weekly  No control group |
| 21 | Lalèyè, F.T.F et al. 2023 | Benin | 289 Children aged 6 to 59 months  **Admission criteria:** WHZ <-2 and ≥-3 (WHO Growth Standards) or MUAC <12.5 cm and ≥11.5 cm | **Group 1:** A multi-ingredient infant flour (FARIFORTI), **LPF (n=289)**  **Group 2:** no control group  **Duration:** 12 days | Weight gain | - The multi-ingredient porridge (FARIFORTI) based on local food resources was preferred and accepted by mothers and children, and it is likely to contribute effectively to weight gain | Included in systematic review only  LPF  No control group |

# **Supplemental Table 4 (A): Cochrane risk of bias of included Randomized Controlled studies**

# **Supplemental Table 4 (B):** **Cochrane risk of bias of included Cluster-Randomized Controlled studies**

# **Supplemental Table 4 (C): Cochrane risk of bias of included non-Randomized Controlled studies**

# **Supplemental Table 5: Pooled effects of locally available foods (LAFs) and locally processed foods (LPFs) on secondary outcomes in the management of moderate acute malnutrition (MAM)**

| **Outcomes measure** | **Interventions** | **Control** | **# studies** | **Pooled effect (95%CI)** | **I² (95%CI)** |
| --- | --- | --- | --- | --- | --- |
| WHZ change | LAFs | Overall | 3 | -0.28 [-0.44, -0.11]* | 0.0 [0.0, 0.0] |
|  |  | LPFs | 1 | -0.34 [-0.56, -0.12]* | NA |
|  |  | RUSF | 1 | -0.20 [-0.53, 0.13] | NA |
|  |  | FBF | 1 | -0.20 [-0.54, 0.14] | NA |
|  | LPFs | Overall | 7 | -0.04 [-0.24, 0.15] | 93.8 [89.6,96.3] |
|  |  | FBF | 4 | 0.04[-0.17, 0.25] | 92.1[82.9, 96.3] |
|  |  | RUSF | 3 | -0.15 [-0.47, 0.16] | 91.7[78.8?96.7] |
| MUAC gain | LAFs | Overall | 7 | −0.16 [−0.37, 0.04] | 93.6 [89.2, 96.2] |
|  |  | FBF | 4 | −0.06 [−0.34, 0.23] | 96.1 [92.6, 97.9] |
|  |  | RUSF | 3 | −0.34 [−0.54,-0.14] | 77.2 [25.9, 93.0] |
|  | LPFs | Overall | 5 | -0.18 [-0.33, -0.04]* | 82.9 [61.1, 92.6] |
|  |  | FBF | 2 | -0.15 [-0.25, -0.05]* | 0.0 [0.0, 0.0] |
|  |  | RUSF | 3 | -0.21 [-0.45, 0.04] | 90.4 [74.7, 98.4] |
| Height gain | LAFs | Overall | 8 | -0.21 [-0.33, -0.09]* | 38.3 [0.0,72.7] |
|  |  | LPFs | 1 | -0.26 [-0.64, 0.12] | NA |
|  |  | RUSF | 3 | -0.15 [-0.33, 0.03] | 0.0 [0.0, 89.8] |
|  |  | FBF | 4 | -0.25 [-0.58, 0.07] | 71.3 [18.3,89.9] |
|  | LPFs | Overall | 6 | -0.15 [-0.30,-0.01]* | 81.6 [60.8,91.4] |
|  |  | RUSF | 3 | -0.27 [-0.39, -0.14]* | 0.0 [0.0, 84.7] |
|  |  | FBF | 3 | 0.05 [0.02, 0.08]* | 73.9 [0.0, 94.1] |

LAF-Locally available foods, LPF-Locally processed foods, SNF-Specialized Nutritious Food, MUAC (Mid-Upper Arm Circumference), WHZ change (Weight-for-Height Z-score change), RUSF: Ready-to-Use Supplementary Food and FBF: Food-Based Foods, "NA" for "Not Applicable", * statistically significant

**Forest plot of different outcomes for LAF and LPF intervention for different outcomes**

A) LAFs-Height

B) LPFs-Height

**Figure S1. Forest plot for the mean difference in height gain between LAFs (A) and LPFs (B) compared to SNFs**

A) **LAFs- MUAC gain**

B) **LPFs-MUAC gain**

# **Supplemental Figure 2. Forest plot for the mean difference in MUAC gain between LAFs (A) and LPFs (B) compared to SNFs**

A) **LAFs-deterioration rate**

# **Supplemental Figure 3. Forest plot for the risk difference in deterioration rate LAFs (A) compared to SNFs**

A) **LAFs-death rate**

# **Supplemental Figure 4. Forest plot for the risk difference death rate in LAFs (A) compared to SNFs**

A) **LAFs-WHZ at discharge**

B) **LPFs-WHZ at discharge**

# **Supplemental Figure 5. Forest plot for the mean difference in WHZ change at discharge for A) LAFs and B) LPFs compared to SNFs**

**Sensitivity analysis**

**A)**

B)

**Supplemental Figure 6: Sensitivity analysis for A) recovery rate for LAFs and LPFs and B) weight gain for LAFs and LPFs compared to standard nutritional foods (SNFs) using the leave-one-out procedure**

**Figure S6 Legend:** The figure presents sensitivity analyses assessing the robustness of pooled estimates for four key outcomes weight gain and recovery rate, in comparisons of alternative food interventions with SNFs. In each panel (A–B), one study at a time was excluded (leave-one-out method), and the pooled effect was recalculated. The stability of results across iterations indicates that no single study disproportionately influenced the overall findings.

**Publication bias**

A)

B)

**Supplemental Figure 7: Contour-enhanced funnel plots for weight gain in the LAFs (A) and LPFs (B) intervention groups.**

A)

B)

# **Supplemental Figure 8: Contour-enhanced funnel plots for Recovery rate in the LAFs (A) and LPFs (B) intervention groups.**

# **Supplemental Figure S9: Contour-enhanced funnel plots for height gain in the LAFs (A) and LPFs (B) intervention groups.**

# **Supplemental Figure S10: Contour-enhanced funnel plots for WHZ change in the LAFs intervention groups**

A)

B)

# **Supplemental Figure S11: Contour-enhanced funnel plots for MUAC in the LAFs (A) and LPFs (B) intervention groups.**
